# Integration of sRNA, degradome, transcriptome analysis and functional investigation reveals gma-miR398c negatively regulates drought tolerance via *GmCSDs* and *GmCCS* in transgenic Arabidopsis and soybean

**DOI:** 10.1186/s12870-020-02370-y

**Published:** 2020-05-05

**Authors:** Yonggang Zhou, Weican Liu, Xiaowei Li, Daqian Sun, Keheng Xu, Chen Feng, Idrice Carther Kue Foka, Toi Ketehouli, Hongtao Gao, Nan Wang, Yuanyuan Dong, Fawei Wang, Haiyan Li

**Affiliations:** College of Life Sciences, Engineering Research Center of the Chinese Ministry of Education for Bioreactor and Pharmaceutical Development, Jilin Agricultural University, Changchun, 130118 Jilin China

**Keywords:** miRNA, Degradome, Transcriptome, Gma-miR398c, Drought, Soybean

## Abstract

**Background:**

Drought conditions adversely affect soybean growth, resulting in severe yield losses worldwide. Increasing experimental evidence indicates miRNAs are important post-transcriptional regulators of gene expression. However, the drought-responsive molecular mechanism underlying miRNA–mRNA interactions remains largely uncharacterized in soybean. Meanwhile, the miRNA-regulated drought response pathways based on multi-omics approaches remain elusive.

**Results:**

We combined sRNA, transcriptome and degradome sequencing to elucidate the complex regulatory mechanism mediating soybean drought resistance. One-thousand transcripts from 384 target genes of 365 miRNAs, which were enriched in the peroxisome, were validated by degradome-seq. An integrated analysis showed 42 miRNA–target pairs exhibited inversely related expression profiles. Among these pairs, a strong induction of gma-miR398c as a major gene negatively regulates multiple peroxisome-related genes (*GmCSD1a/b, GmCSD2a/b/c* and *GmCCS*). Meanwhile, we detected that alternative splicing of *GmCSD1a/b* might affect soybean drought tolerance by bypassing gma-miR398c regulation. Overexpressing gma-miR398c in *Arabidopsis thaliana* L. resulted in decreased percentage germination, increased leaf water loss, and reduced survival under water deficiency, which displayed sensitivity to drought during seed germination and seedling growth. Furthermore, overexpressing gma-miR398c in soybean decreased *GmCSD1a/b, GmCSD2a/b/c* and *GmCCS* expression, which weakened the ability to scavenge O_2_^.−^, resulting in increased relative electrolyte leakage and stomatal opening compared with knockout miR398c and wild-type soybean under drought conditions.

**Conclusion:**

The study indicates that gma-miR398c negatively regulates soybean drought tolerance, and provides novel insights useful for breeding programs to improve drought resistance by CRISPR technology.

## Background

Soybean (*Glycine max* L.) is the most important legume crop that provides essential vegetable proteins and oil sources for livestock and humans, while its production is severely constrained by global climatic changes, particularly drought stress [1]. However, the current lack of knowledge regarding the regulatory network underlying soybean responses to drought conditions has restricted an effective management of soybeans productivity in the world. Therefore, characterizing the complex molecular mechanism of drought resistance is a prerequisite for improving soybean yield [2].

In plants, small RNAs play a prominent regulatory role influencing the adaptability to drought stress, especially microRNAs (miRNAs) via degradation or translational inhibition of their target genes [3]. In the past decade, some differentially expressed miRNAs (DEMs) under drought-simulated stress have been filtered by small RNA sequencing (sRNA-seq), mainly in soybean roots [4, 5]. However, only few miRNAs in soybean, e.g. gma-miR394 and gma-miR396, by overexpressing in model plants have been verified to perform an important function in response to drought stress [6, 7]. As known, miR398 participates in stress resistance by regulating the expression of superoxide dismutase (SOD)-related genes in many plants, such as *Arabidopsis thaliana* [8], *Medicago sativa* [9], tomato [10], and *Oryza sativa* [11]. Overexpressing miR398 negatively regulates plant stress tolerance in *Arabidopsis thaliana* and *Nicotiana benthamiana* [12, 13]. Recently, rice osa-miR398b overexpression plants and mutants in *CSD1* and *CSD2* were shown to display enhanced basal defenses by altering expression of multiple SODs, indicating that in different plant species miR398 might play different roles in stress resistance [14, 15]. Overall, our understanding of the roles played by miRNAs under drought stress remains limited, especially for seedlings of soybean.

Usually, miRNA researchers combined differentially expressed genes (DEGs) to analyze the regulatory mechanisms in which miRNAs might be involved. In terms of genetic control of drought tolerance, generating hypotheses of quite complex data is a difficult mission, and the accurate identification of targets is necessary. Degradome sequencing (degradome-seq) has been important for identification of global targets of miRNAs, which has helped to clarify the miRNA regulatory network [16]. Recently, a few studies on the integrated analysis of miRNA, degradome and mRNA sequencing have been published in plants [17, 18]. However, the miRNA-target pairs that displayed negative correlations during *soybean mosaic virus* infection were not obtained using the integrated analysis, probably because of the limitations in the number of miRNA targets and DEGs [19]. Overall, gaining an in-depth understanding of the complex molecular mechanisms of soybean miRNAs in the response to drought stress and strategies for genetic improvement of soybean for drought resistance are still a challenge [20]. Therefore, integration of sRNA, degradome and mRNA sequencing is required to clarify the drought-responsive molecular mechanism to narrow the list of candidate genes, and further functional identification is also indispensable in soybean.

The objective of our study was to combine sRNA-seq, degradome-seq and mRNA-seq data to construct a comprehensive dataset useful for identifying the key regulatory miRNAs-targets network, and carry out its functional analysis in transgenic plants under the simulated drought conditions. In the present study, we validated 1000 transcripts from 384 target genes of 365 miRNAs, which were enriched in the peroxisome, and 42 miRNA–target pairs exhibited inversely related expression profiles. Among these pairs, gma-miR398 exhibited the function of a major gene that negatively regulated multiple peroxisome-related genes (*COPPER-ZINC SUPEROXIDE DISMUTASE 1* and *2*, *GmCSD1a/b, GmCSD2a/b/c*; and *COPPER CHAPERONE FOR SUPEROXIDE DISMUTASE*, *GmCCS*) in soybean subjected to water-deficient conditions. Furthermore, we observed that alternative splicing (AS) of *GmCSD1a/b* also might affect soybean drought tolerance by bypassing gma-miR398c regulation, and a greater number of target genes of gma-miR398c than those detected in other legumes was identified. Overall, we suggest that gma-miR398c may affect the redox status to reduce soybean drought resistance. These results provide novel insights that may be useful in breeding programs to improve the drought resistance of soybean by CRISPR-mediated gene editing.

## Results

### Response of miRNAs to drought-simulated stress by sRNA-seq and degradome-seq

To evaluate the drought–responsive miRNA effects, the 8% polyethylene glycol (PEG) 8000 was used to simulate drought stress conditions on soybean seedlings at the first unifoliate leaves stage. Then, sRNA-seq and degradome-seq assays were performed. (Table S1). We identified 716 miRNAs for all libraries from control and treated plants, of which 546 miRNAs from 208 families were known miRNAs, and 170 miRNAs were novel miRNAs that were predicted using miRDeep-p (Table S2). Among these miRNAs, 117 and 99 drought-responsive DEMs in the leaf samples collected at 6 and 12 h were filtered out, respectively. Meanwhile, 144 and 83 DEMs were detected in the root samples harvested at 6 and 12 h, respectively (Table S3). These results showed that most of the miRNA family members have the similar expression patterns. For instance, the expression levels of gma-miR319a/b/e/f/h/j/k/m and gma-miR1514a/b-3p were significantly up-regulated in leaves. Also, gma-miR156aa/g/z, gma-miR4415a/b and gma-miR159b-3p/c/d expression levels were down-regulated in roots. In addition, several members of the same family exhibited differential expression patterns in the same tissues, for example, drought stress up- and down- regulate respective gma-miR396h and gma-miR396d in leaves. Notably, gma-miR5037c expression was all down-regulated in the leaves and roots, while gma-miR5670b expression was up-regulated in leaves and down-regulated in roots, indicating that miRNAs expression patterns need to be identified in individual tissues.

**Fig. 1:**
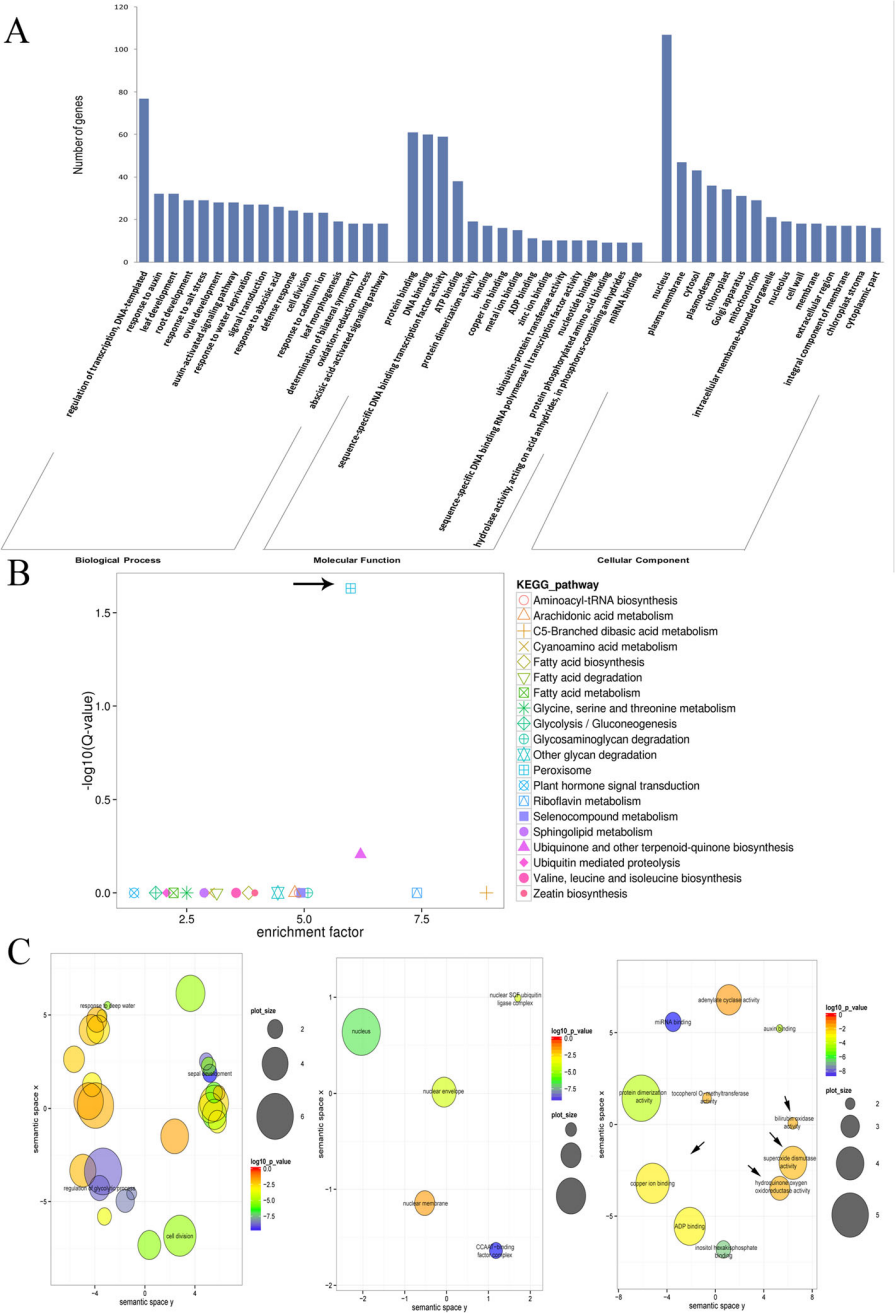
Gene ontology functional classification of identified target genes under water-deficit stress among the degradome-seq libraries. **a** Gene ontology classification of the degraded genes targeted by the identified miRNAs under water-deficit conditions. **b** KEGG analysis of the identified target genes in soybean plants exposed to simulated drought conditions. (**c**) Enrichment of the gene ontology terms associated with the target genes in soybean plants exposed to water-deficit stress. Arrows indicate the important biological pathways

A total of 1000 transcripts from the degradome-seq data belonging to 384 target genes were cleaved by 365 miRNAs. This total included 755 transcripts from 282 genes targeted by 318 miRNAs in leaves, 651 transcripts from 246 genes targeted by 264 miRNAs in roots, and 144 target genes common to both leaves and roots (Table S4). Notably, several GO terms of 384 target genes may be associated with the response to the simulated drought conditions, including response to water deprivation, response to salt stress, defense response, and oxidation–reduction process (Fig. 1a). The Kyoto Encyclopedia of Genes and Genomes (KEGG) analysis revealed that soybean miRNAs could participate in multiple biological pathways through the regulation of target genes, including Peroxisome (ko04146), Ubiquinone and other terpenoid-quinone biosynthesis (ko00130), Aminoacyl-tRNA biosynthesis (ko00970), and Glycine, serine and threonine metabolism (ko00260). Among these pathways, the peroxisome pathway was significantly enriched, and included *SOD* and *LONG-CHAIN ACYL-COA LIGASE* (*ACSL*) genes (Fig. 1b). Analysis of GO term enrichment (Fisher’s test, *P* < 0.05) using the REVIGO software revealed a number of significantly enriched GO terms, including superoxide dismutase activity (GO:0004784), bilirubin oxidase activity (GO:0047705), hydroquinone:oxygen oxidoreductase activity (GO:0052716), and copper ion binding (GO:0005507) (Fig. 1c). The above analyses indicated that soybean miRNAs considerably participate in drought response, especially peroxisome pathway that plays a key role in redox signalling and lipid homeostasis.

### Correlation analysis of sRNA-seq, degradome-seq, and mRNA-seq between miRNAs and target genes

**Fig. 2:**
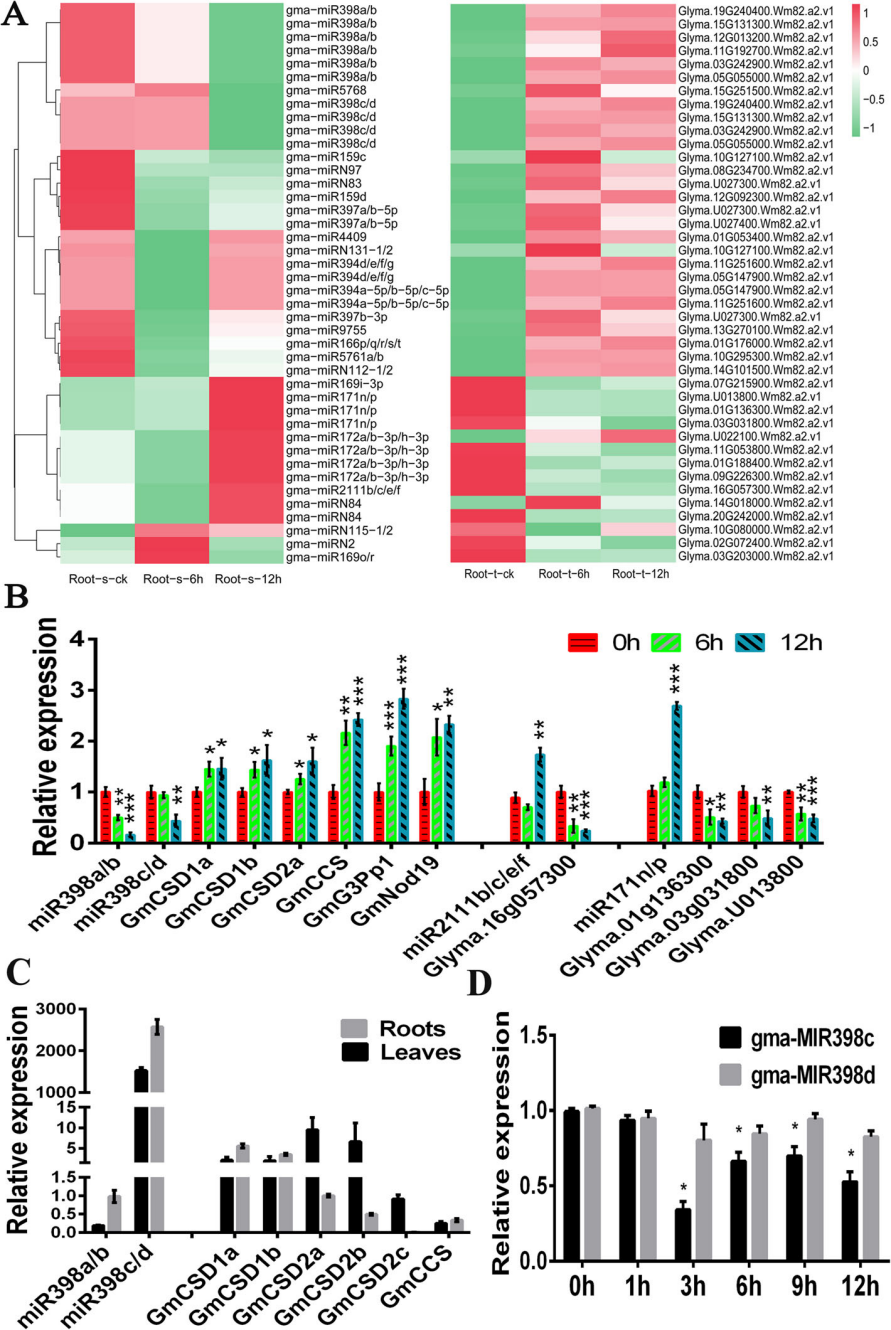
Comparison of expression levels between differentially expressed miRNAs and their target genes. **a** Comparison of expression levels between differentially expressed miRNAs and their target genes in soybean roots based on sRNA-seq, degradome-seq and mRNA-seq data. The original expression values underwent a Z-score normalization; normalized signal values = log_10_(FPKM). **b** Results of a RT-qPCR confirmation of three miRNAs and their target genes identified in sequencing data. Expression levels of each gene in soybean plants cultured under normal conditions served as the controls. **c** Drought stress-induced tissue-specific expression of miR398s and their targets in soybean plants. Expression analyses of mature gma-miR398s and their targets in soybean leaves and roots cultured under normal conditions. Expression levels of mature gma-miR398a/b and *GmCSD2a* in roots served as controls. (D) Expression of gma-MIR398c and gma-MIR398d during water deficit. Total RNA samples obtained from 8% PEG treated or control soybean roots for 1, 3, 6, 9 or 12 h were used to determine precursor gma-miR398c and precursor gma-miR398d abundance by RT-qPCR. Values are average of three biological replicates ± SD, different letters and asterisks indicate significant difference applying ANOVA (*, *P <* 0.05; **, *P <* 0.01; ***, *P <* 0.001)

In order to investigate the miRNAs-mediated drought-response mechanism, transcriptome sequencing was implemented to provide a reliable complement to improve the accuracy of the analysis (Table S1). Firstly, the genomic positions and annotations of the known and novel genes were clarified (Table S5). Continuously, a total of 2 and 55 DEGs at 6 and 12 h were detected for leaf samples, and 6075 and 6761 DEGs were detected at 6 and 12 h for root samples (Table S6). After that, an integrated analysis of sRNA, mRNA, and degradome data in roots and leaves identified 922 and 789 pairs, respectively, of negatively regulated miRNA targets. Amongst these pairs, the reverse expression of only 42 pairs in roots changed significantly more than 1.5-fold (Fig. 2a). For instance, gma-miR398s negatively regulated the expression of multiple genes (e.g., *GmCSD1a*, *GmCSD1b*, *GmCSD2a*, *GmCCS*, *GmNod19* and *GmG3Pp1*) related to inorganic ion transport and metabolism, stress up-regulated Nod 19 and glycerol-3-phosphate transporter 1. The expression levels of Glyma.01G136300, Glyma.03G031800 and Glyma.U013800, which were annotated as scarecrow-like proteins, were regulated by gma-miR171n/p. Additionally, Glyma.16G057300, which was annotated as the F-box/kelch-repeat protein-like At3g27150, was regulated by gma-miR2111b/c/e/f. Furthermore, the expression levels of 10 interesting miRNA targets were analysed to confirm that the genes were negatively regulated by miRNAs, which produced similar expressional patterns with the sequencing results (Fig. 2b). Meanwhile, the KEGG analysis indicated that the soybean drought-responsive genes regulated by miRNAs were mainly involved in peroxisome pathway (ko04146) (Fig. 1B), which included four DEGs (*GmCSD1a/b*, *GmCSD2a*, and *GmCCS*) associated with *GmSOD*-related genes. Overall, correlation analyses also suggested that gma-miR398 might function as a major gene that negatively regulated multiple peroxisome-related genes in soybean plants exposed to simulated drought conditions (Figure S1).

### Detection of expression patterns and interaction between miR398 and its target genes

Identification of the characteristics of gma-miR398 and its targets in soybean was a prerequisite to understand the multiple interactions of the miR398-targets module. Since the gma-miR398 family members were conserved, we considered that their targets might be simultaneously cleaved by gma-miR398a/b and gma-miR398c/d (Figure S2A). Firstly, we observed that gma-miR398c/d expression levels were much higher than those of gma-miR398a/b in leaves and roots (Fig. 2c). Meanwhile, the abundance of the precursor gma-miR398c (gma-MIR398c) was inhibited more distinctly than that of gma-MIR398d in response to simulated drought stress, thus gma-miR398c might play a more important role in drought resistance in soybean (Fig. 2d). Secondly, no differences in the expression of *GmCSD1a/b* were observed between roots and leaves, whereas *GmCSD2a/b/c* expression levels were substantially higher in leaves than in roots, which clearly exhibited tissue-specific expression patterns (Fig. 2c). These results provide evidence that gma-miR398c might play an important role in the regulation of peroxisome-related pathways.

With an aim to understand the molecular mechanisms of the interaction between gma-miR398c and target genes, a modified 5′ RACE method and a transient GFP-dependent gene expression method were performed to verify the degradome-seq data. The degradome-seq results revealed that *GmCSD1a/b*, *GmCSD2a*/*b*, and *GmCCS* were regulated by gma-miR398c, while G*mCSD2c* was not listed (Figure S1). Owing to that the interaction sites between gma-miR398c and the three *GmCSD2a/b/c* genes were conserved, we considered that G*mCSD2c* was also a potential target for gma-miR398c. Firstly, the targets of gma-miR398c were validated using a modified 5′ RACE method. Among the above-mentioned genes, *GmCSD1a*/*b*, *GmCSD2a*, and *GmCCS* were directly identified (Figure S3). However, two *GmCSD2b/c* were not detected using 5′ RACE, probably because of their relatively low expression levels or translation repression. Notably, absence of the miRNA binding sites (MBSs) of *5′UTR-GmCSD1a-1* and *5′UTR-GmCSD1b-1* prohibited gma-miR398c regulation, whereas transcripts of *GmCSD1* contained the MBS (Figure S2B), which probably affected the regulation of *GmCSD1a/b* expression under abiotic stress [21].

**Fig. 3:**
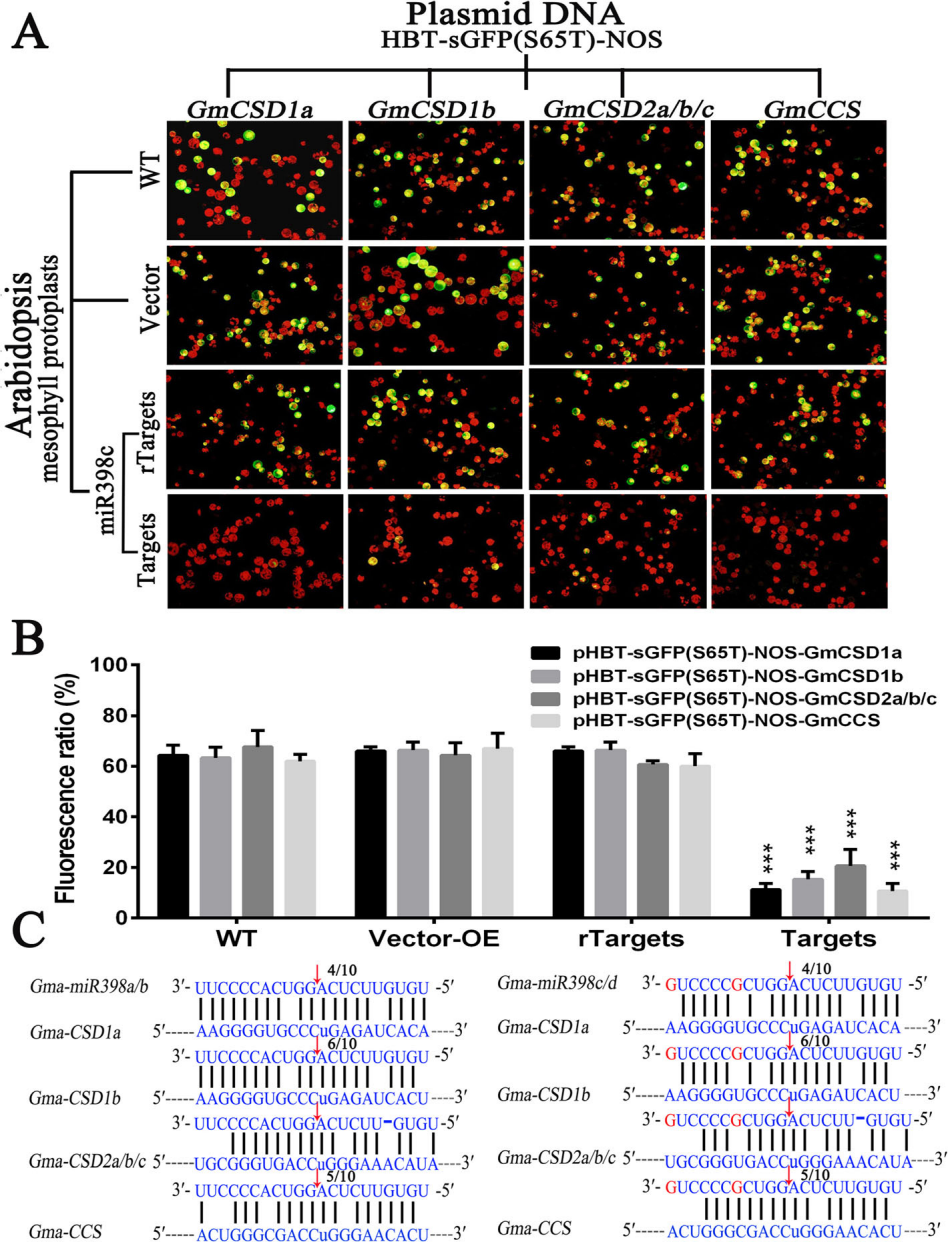
Interaction between gma-miR398c and its targets verified in transiently transformed mesophyll protoplasts. **a** Interaction vector [HBT-sGFP(S65T)-NOS–Targets] or [HBT-sGFP(S65T)-NOS–rTargets] inserted into mesophyll protoplasts derived from WT, OE-vector and OE-miR398c transgenic Arabidopsis plants. Bar = 50 μm; (**b**) Fluorescence ratio of mesophyll protoplast cells. Values are average of three replicates ± SD. Asterisks indicate significant difference applying Student’s t test (*, *P <* 0.05; **, *P <* 0.01; ***, *P <* 0.001). **c** Details regarding the gma-miR398c cleavage site in the target genes (*GmCSD1a*, *GmCSD1b*, *GmCSD2a/b/c* and *GmCCS*)

Based on the results above, the fluorescence ratios in Arabidopsis mesophyll protoplasts generated by a transient GFP-dependent gene expression method were evaluated to further characterize the interactions between miRNAs and their targets. Firstly, gma-miR398c overexpression plants (OE-miR398c) were selected as a generator of gma-miR398c, wild-type Arabidopsis (WT) and empty vector overexpression plants (OE-vector) were used as negative control. Secondly, we inserted the HBT-sGFP(S65T)-NOS–Targets plasmids (Targets) into Arabidopsis mesophyll protoplasts prepared from WT, OE-vector and OE-miR398c plants, and the HBT-sGFP(S65T)-NOS–rTargets plasmids (rTargets) were transformed into OE-miR398c protoplasts as a positive control (Fig. 3a). The results displayed that the fluorescence ratios of HBT-sGFP(S65T)-NOS–Targets from OE-miR398c plants were considerably lower than those in the WT, OE-vector, or rTargets plants (Fig. 3b). Therefore, these data confirmed that gma-miR398c could negatively regulate *GmCSD1a/b*, *GmCSD2a/b/c*, and *GmCCS* (Fig. 3c)*.*

### Subcellular localization of GmCSDs and GmCCS proteins

**Fig. 4:**
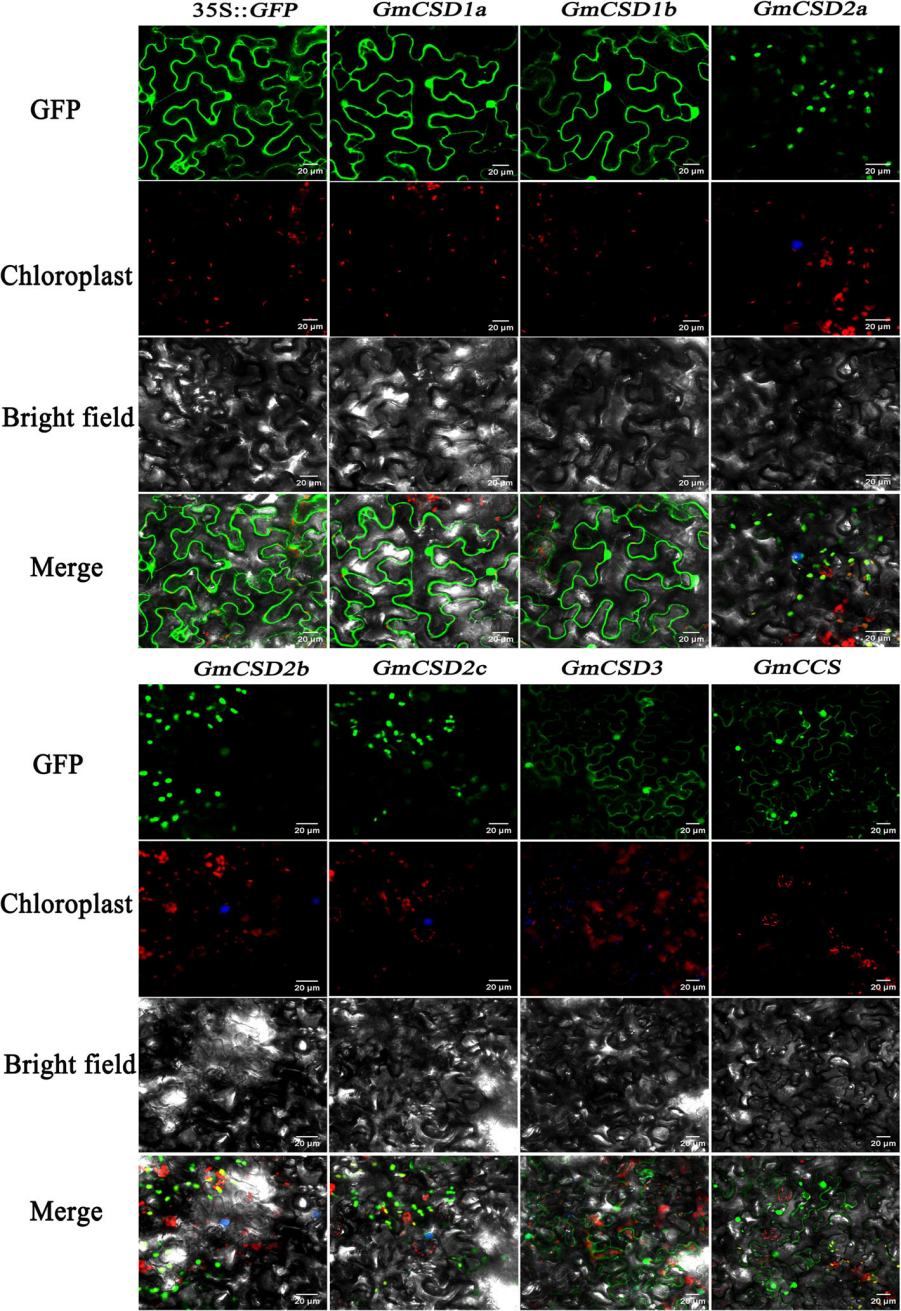
Subcellular localization of GmCSDs and GmCCS in tobacco mesophyll cells. Tobacco epidermal cells expressing 35S:GmCSDs-GFP, 35S:GmCCS-GFP, or 35S:GFP were separately viewed by a confocal microscope. The nucleus marker (NLS) and peroxisome marker (SKL) indicated by the blue fluorescence were separately used to co-localize with GmCSD2a/b/c and GmCCS. Bar = 20 μm

As known, copper-zinc superoxide dismutases (CSDs) are the predominant scavengers of O_2_^.−^ to maintain the ROS balance with the influence of CCS [22]. However, previous research has mainly focused on the proteins and changes in activity of their targets, as the expression and function of these genes in soybean remains poorly investigated [23]. Therefore, the subcellular localizations of GmCSDs and GmCCS were examined for further functional characterization of these genes. Compared with signals of the empty vector in transient transformation assays in Arabidopsis mesophyll protoplasts, signals of GmCSD1a/b were observed in the cytoplasm, GmCSD2a/b/c and GmCCS signals were predominantly found in the chloroplasts, and no GmCSD3 signals were detected (Figure S4). For the further verifications, GmCSDs and GmCCS were transiently expressed in tobacco leaves that contained NLS and SKL markers (Fig. 4). Signals for GmCSD1a/b were detected in the cytoplasm and nucleus, and those for GmCCS were localized in the chloroplasts, nucleus, and cytoplasm. Signals for GmCSD2a/b/c co-localized with NLS were detected in the nucleus and chloroplasts, which was consistent with the predominant expression of these genes in the leaves of soybean (Fig. 2c). The co-localization of GmCSD3 with SKL showed that GmCSD3 was expressed in the peroxisome, nucleus, and cytoplasm. Taken together, these results strongly suggested that the gma-miR398c-targeted *GmCSDs* and *GmCCS* genes might play an important role in elimination of O_2_^.−^ in cells key organelles.

### Phenotypic analysis of OE-miR398c transgenic Arabidopsis under simulated drought conditions

**Fig. 5:**
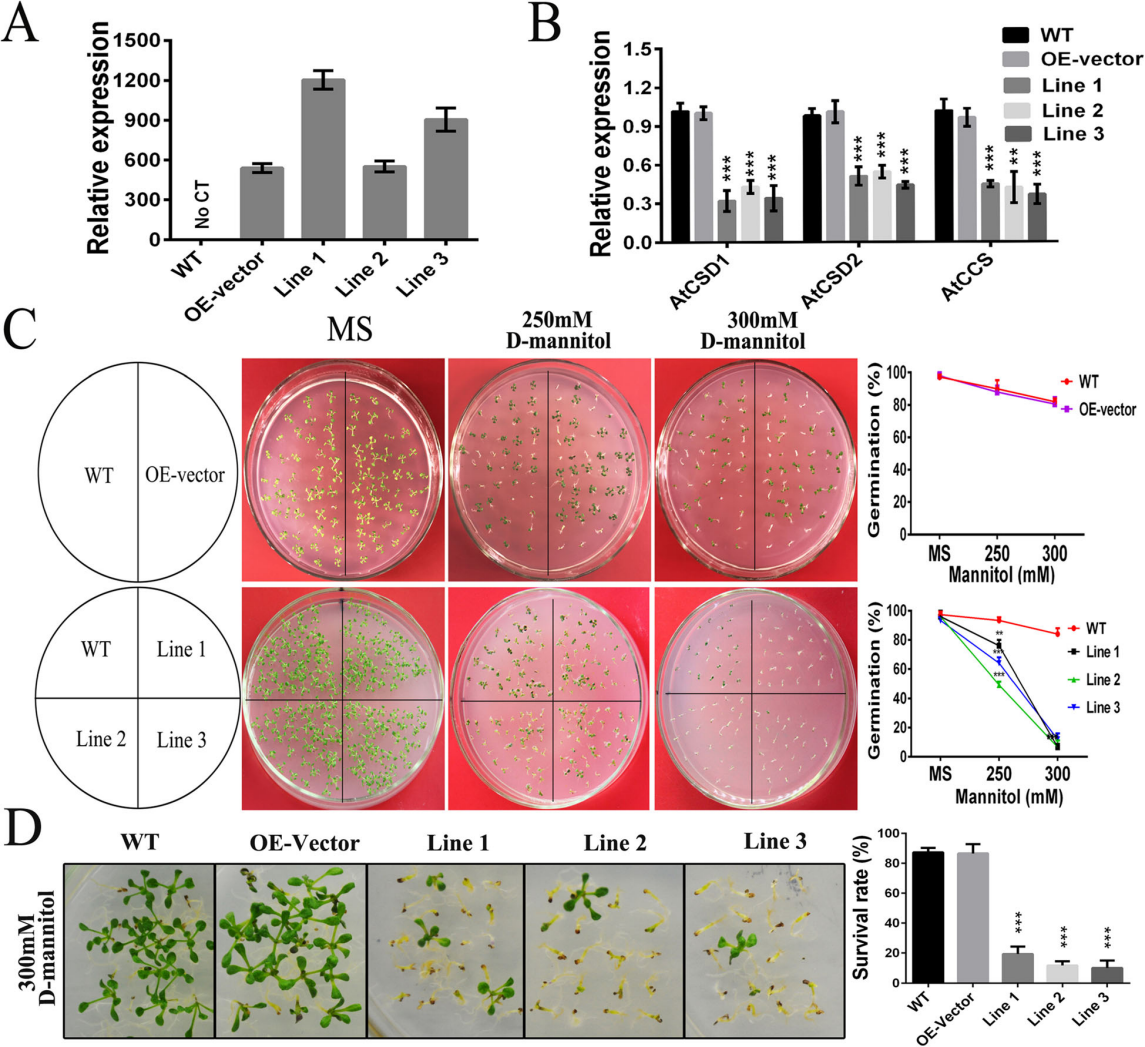
Responses of transgenic plants to simulated drought conditions. **a, b** Expression of the gma-miR398c precursor (gma-MIR398c) and the relative expression of ath-miR398c target genes in transgenic Arabidopsis lines determined by RT-qPCR. Expression levels were normalized against that of the *Actin* gene. **c** Seeds of WT, OE-vector and OE-miR398c were germinated on plates containing different concentrations of mannitol. Germination rates were analyzed after 14 days. **d** Survival rate of Arabidopsis seedlings after 10 days cultivation on 300 mM D-mannitol medium**.** Vertical bars represent the standard deviation of the means (*n* = 3, 200 seeds per replicate). Asterisks indicate significant differences between the transgenic and WT lines (*, *P <* 0.05; **, *P <* 0.01; ***, *P <* 0.001)

**Fig. 6:**
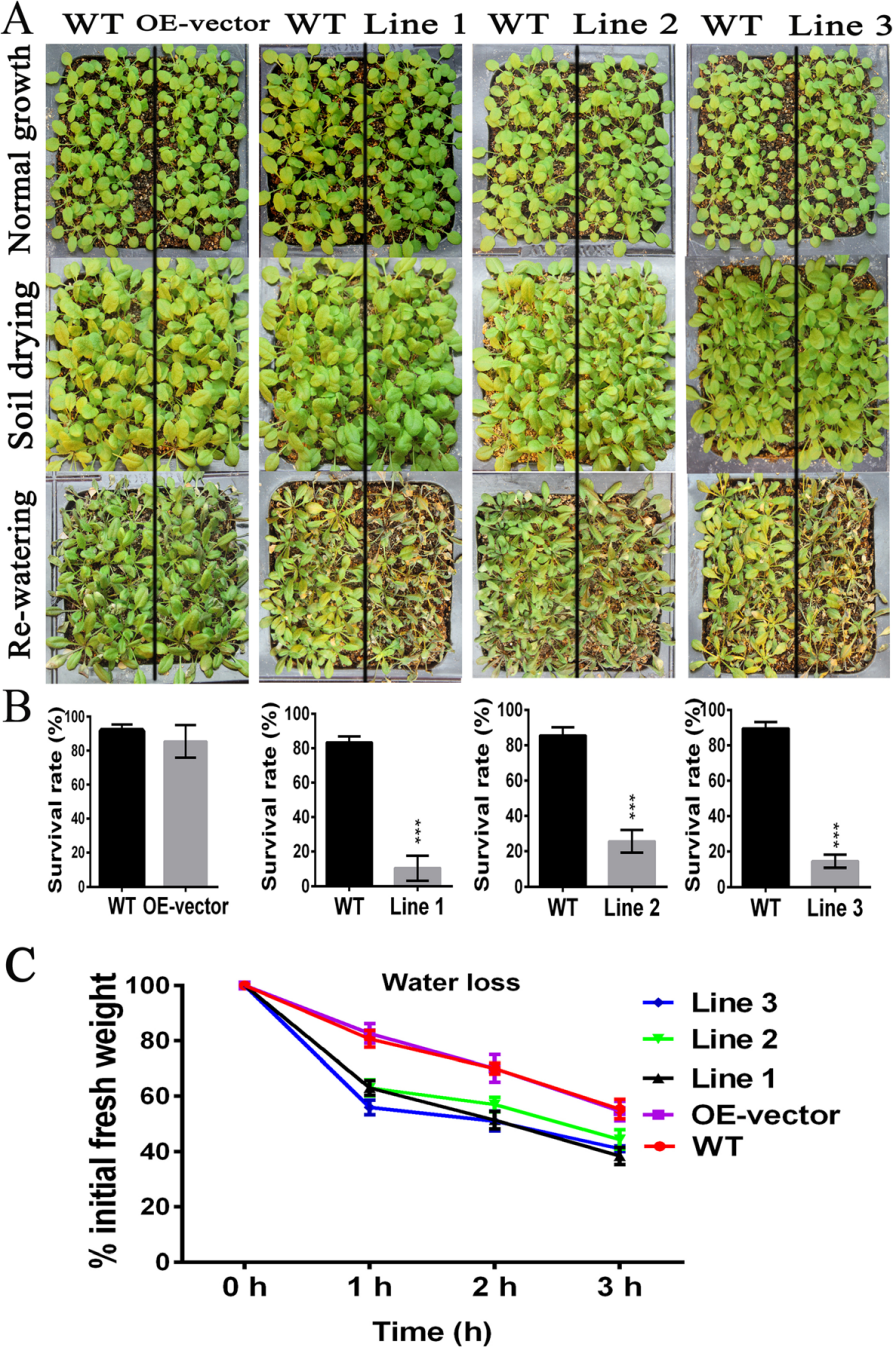
Analyses of the drought tolerance of OE-miR398c transgenic Arabidopsis seedlings. **a** Representative images of WT, OE-vector and OE-miR398c Arabidopsis plants at three stages (normal growth, soil drying and re-watering). **b** Seedling survival rates of OE-miR398c transgenic Arabidopsis plants grown in soil under drought conditions. **c** Water loss rate of WT and transgenic Arabidopsis*.* Detached leaves were incubated on a bench, and the fresh weight was measured at different durations. Data are presented as the average of five replicates ± standard deviation (treatments with 25 seedlings). Asterisks indicate significant differences between the transgenic and WT lines (***, *P <* 0.001)

To evaluate whether overexpression of gma-miR398c affected Arabidopsis drought tolerance, a seed germination assay on 1/2 MS medium supplemented with D-mannitol (0, 250, or 300 mM) was performed. The expression levels of gma-MIR398c and the *bar* gene were distinctly up-regulated in transgenic lines and were not detected in WT plants (Fig. 5a). In contrast, the *AtCSD1*, *AtCSD2*, and *AtCCS* expression levels were lower in the transgenic plants than in the WT plants (Fig. 5b). The seed germination rates on MS medium (0 mM D-mannitol) were similar in the WT, OE-vector, and OE-miR398c plants. Non-significant differences in percentage seed germination of the WT and OE-vector lines was observed on MS medium supplemented with 250 or 300 mM D-mannitol, whereas the germination frequency and survival rates for the OE-miR398c lines was significantly reduced (Fig. 5c, d). In addition, we calculated the percentage survival of plants grown in soil under simulated drought as well as the evaluation of leaf water loss rate. The survival rates of OE-miR398c lines were considerably lower than those of WT and OE-vector plants under the simulated drought condition (Fig. 6a, b). The water-loss rate was higher for detached leaves from OE-miR398c lines than for those from OE-vector or WT plants under the water-deficient condition (Fig. 6c). These results indicated that overexpression of gma-miR398c decreased the drought tolerance of transgenic Arabidopsis plants.

### Characterization of gma-miR398c function in soybean

**Fig. 7:**
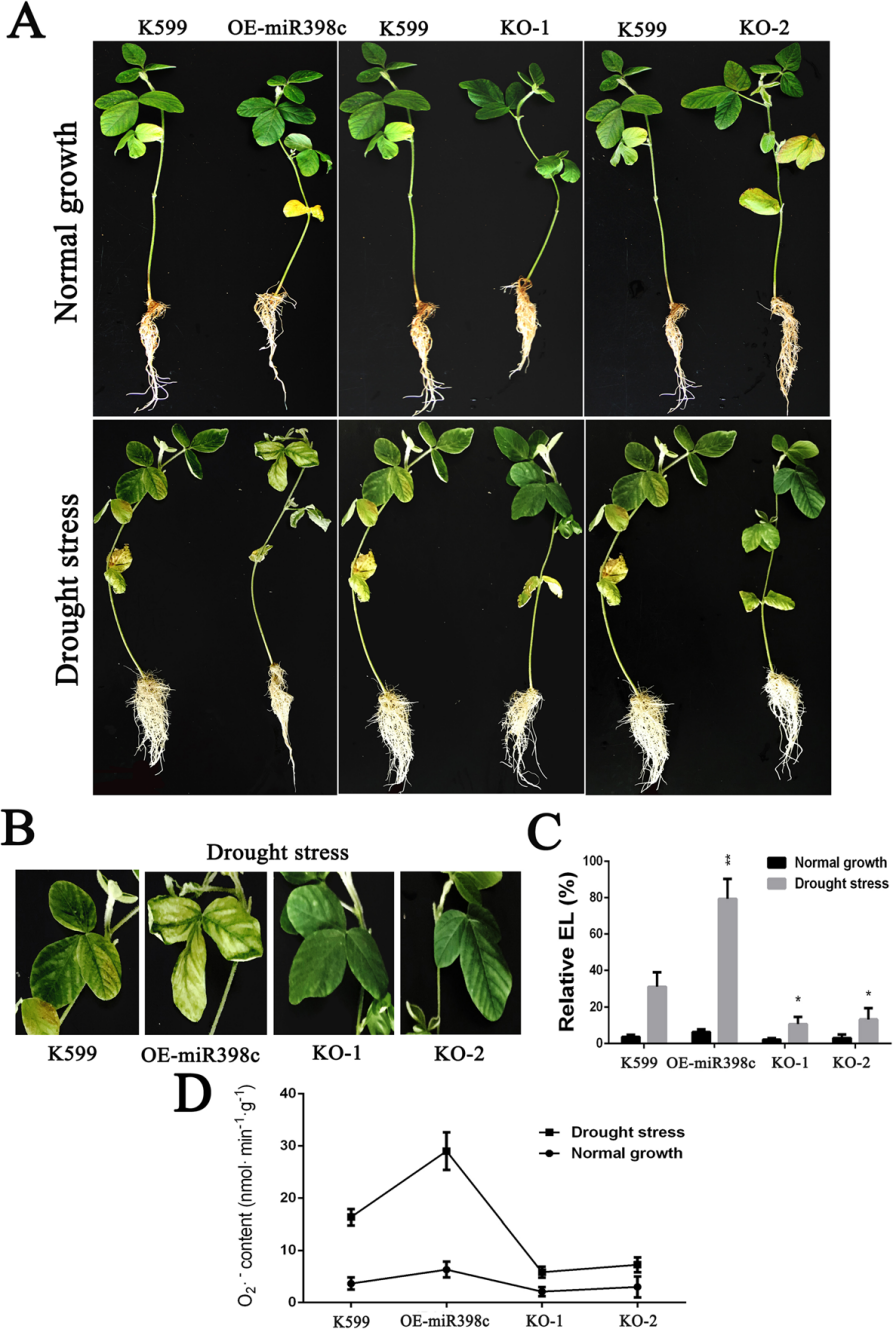
Effects of knocking out and overexpression of gma-MiR398c composite soybean plants on drought resistance. **a** Performance of soybean with K599, OE-miR398c, KO-1 and KO-2 transgenic roots after drought stress. **b** Enlargement of the leaves from the soybean plants. **c** Relative electrolyte leakage in leaves from the soybean with transgenic hairy roots compared to the normal K599. (**d**) O_2_^.−^ production rate in plant leaves. Control K599 represents hairy roots transformed with the empty vector; OE indicates transgenic hairy roots overexpressing gma-MIR398c; KO indicates transgenic hairy roots harboring the pCas9-U6-sgRNAs plasmid. Data are means of ten biological replicates ± SD (10 plants per replicate). Asterisks indicate significant difference applying Student’s t test (*, *P <* 0.05; **, *P <* 0.01; ***, *P <* 0.001)

**Fig. 8:**
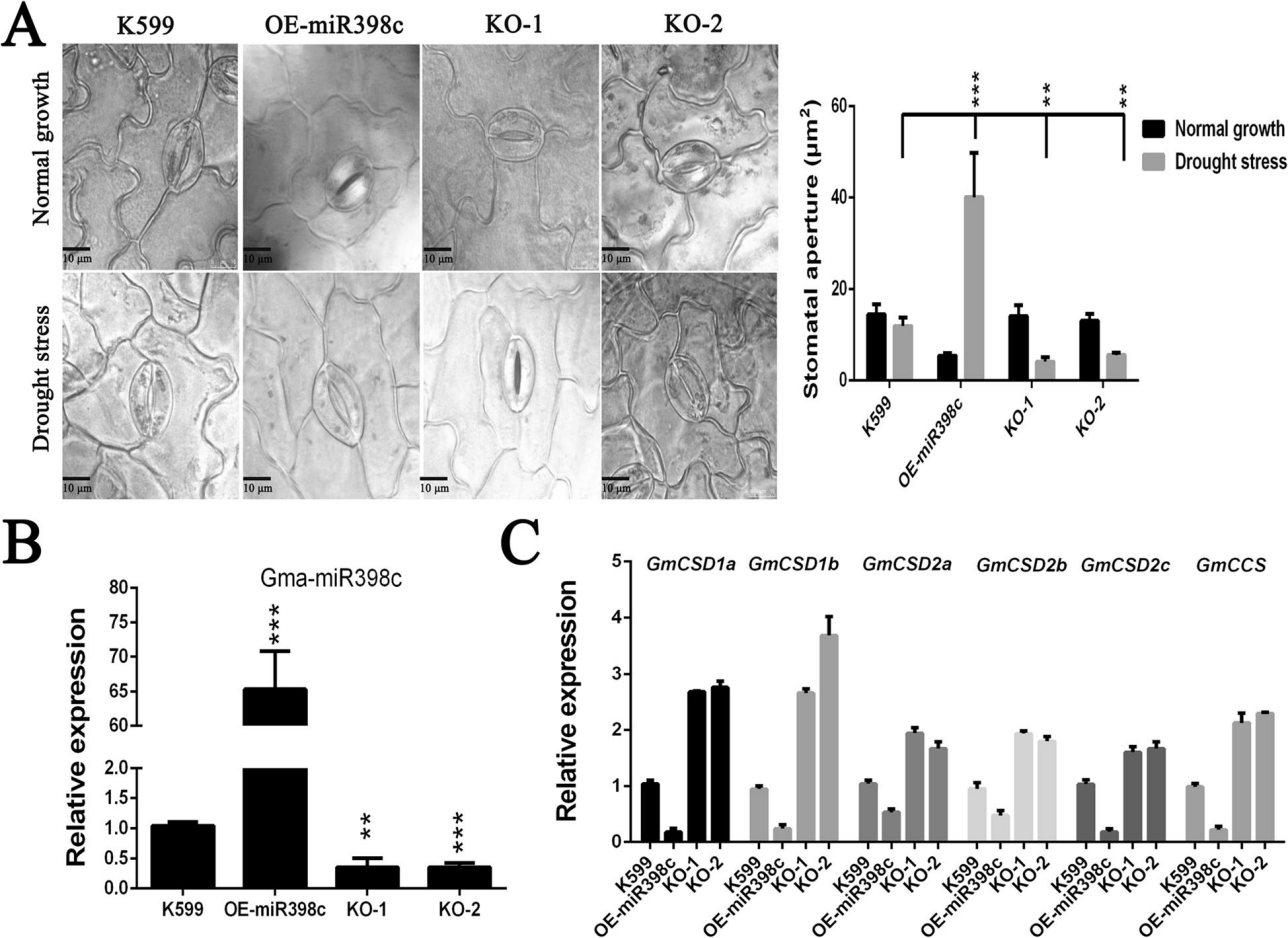
Effect stomatal aperture and gene expression for gma-miR398c knockout and overexpression in composite soybean plants. **a** Analysis of stomatal aperture of leaves before and after drought stress for soybean plants. The second tri-foliate from the top were chosen for stoma observation. Data are average of 30 views ± SD, asterisks indicate significant difference applying ANOVA between drought treated K599 and transgenic plants (*, *P <* 0.05; **, *P <* 0.01; ***, *P <* 0.001). Bar = 10 μm. **b** Analysis of miR398c gene expression in K599, OE-miR398c, KO-1 and KO-2 transgenic soybean hairy roots. **c** Analysis of *GmCSD1a/b, GmCSD2a/b/c* and *GmCCS* expression in K599, OE-miR398c, KO-1 and KO-2 transgenic soybean hairy roots. Data are means of ten biological replicates ± SD (10 plants per replicate)

To further assess whether gma-miR398c in soybean may participate in drought regulation mechanisms, *Agrobacterium rhizogenes*-mediated transformation of soybean hairy roots with edited gma-MIR398c was performed to evaluate gma-miR398c function. The wilting of OE-miR398c leaves was severer than that of the other lines, whereas the leaves of KO-1 and KO-2 plants grew better than control plants (Fig. 7a, b). To evaluate the degree of membrane damage in these leaves, the relative electrolyte leakage and O_2_^.−^ content were measured. The leaves from OE-miR398c plants showed distinctly higher relative electrolyte leakage and O_2_^.−^ production than the leaves of the other lines (Fig. 7c, d). Considering that O_2_^.−^ production could affect stomatal closure, we investigated the possible responsible mechanisms. Stomatal aperture measurements showed that stomatal opening of OE-miR398c plants was significantly increased compared with that of control seedlings after stress for 3 h. In contrast, stomatal opening of KO-1 and KO-2 plants was reduced, which was consistent with the leaf O_2_^.−^ contents (Fig. 8a). Previous studies indicated that CSDs played a predominant antioxidant role to scavenge O_2_^.−^, compared with other SODs, under abiotic stress [24, 25]. Then, the expression levels of these genes were analysed by RT-qPCR in the soybean hairy roots. Our results displayed that the expression of gma-miR398c in the hairy roots of OE-miR398c plants was significantly increased, whereas the expressions of *GmCSD1a/b*, *GmCSD2a*/*b*, and *GmCCS* were distinctly inhibited compared with those of the control plants. In contrast, the expression level of gma-miR398c in KO-1 and KO-2 plants was reduced, and the expressions of its target genes were enhanced (Fig. 8b, c). These results indicated that gma-miR398c considerably affected the redox status to reduce the drought resistance of soybean by altering the expression levels of multiple *GmCSD1a/b*, *GmCSD2a*/*b*, and *GmCCS* genes.

## Discussion

Reprogramming the drought-responsive miRNAs to activate specific target genes is an important molecular mechanism involved in the evolution of drought resistance in plants. The water deficiency status in plants is an important factor associated with drought stress [26]. Previously, sRNA-seq and mRNA-seq have been used separately to investigate the effects of simulated drought stress on soybean plants, and to detect DEMs and DEGs or gene regulatory networks potentially involved in the stress response [5, 27, 28]. However, the molecular mechanism underlying the miRNA–mRNA interactions under water-deficient conditions in soybean remained largely unknown. Thus, we undertook a comprehensive quantitative and qualitative analysis of the complex regulatory mechanisms induced by water deficiency and detected the function of candidate genes. Our findings provide valuable insights to guide future studies on unique regulatory links between miRNAs and their target genes. In the present study, most miRNAs of the same family exhibited similar significantly altered expression patterns in response to water deficiency. For example, gma-miR408ac/b-5p were all down-regulated in roots, and miR319 family members were up-regulated in leaves, which was consistent with previous results [29, 30]. However, Zheng et al. [5] reported that simulated drought stress up-regulated miR2111 expression, while we observed that gma-miR2111b/c/e/f were up-regulated in leaves, but were down-regulated in roots. Indeed, it is likely that the same miRNA in the same plant species under different externally applied concentrations of PEG displayed differential expression [31]. In addition, gma-miR5037c was down-regulated in soybean leaves and roots under simulated drought conditions, while Chen et al. [19] reported that miR5037c was significantly up-regulated in soybean mosaic virus-infected leaves. These results imply that drought-responsive miRNAs might participate in biotic stress by different expression patterns, that leads to the corresponding change in expression patterns of miRNA targets. Therefore, it is necessary to determine the exact miRNA functions by analyzing the regulatory network of target genes.

### Integration of sRNA-seq, degradome-seq, and mRNA-seq revealed gma-miR398c as a major gene involved in peroxisome

Previous studies indicated that drought treatment mainly induced accumulation of two specific types of ROS, namely H_2_O_2_ and O_2_^.−^, resulting in oxidative stress and cell damage [20], which was consistent with GO enrichment and KEGG analysis of soybean miRNA targets from our degradome-seq data. Thus, determining how to utilize soybean miRNAs to activate additional target genes involved in plant defense against oxidative stress will be important for maintenance of ROS homeostasis [32]. Here, we determined that 42 miRNA–target pairs exhibited inversely related expression profiles in roots by integrating the data from sRNA-seq, degradome-seq, and mRNA-seq analyses. Meanwhile, we observed that down-regulated gma-miR398c displayed the function of a major gene that negatively regulated the expression of multiple peroxisome-related genes (e.g., *GmCSD1a*/*b*, *GmCSD2a*, and *GmCCS*) (Fig. 2b). The enzymes encoded by these target genes represent the first protective barrier that efficiently eliminates free radicals and prevents plants from accumulating ROS to toxic levels [33]. Recent studies have shown that miR398 may positively regulate rice stress tolerance via altering the expression of CSDs to improve total SOD enzyme activity, which is different from its negative role in defense in Arabidopsis, thus it is particularly important to analyse the function of gma-miR398c under drought stress in soybean [14, 15].

### Drought-responsive accumulation of *GmCSDs* and *CCSs* are affected by gma-miR398c manipulation in soybean

To understand the interaction network of miR398–CSD–CCS module in soybean responses to water deficit, a modified 5′ RACE method and a transient GFP-dependent gene expression assay were performed to identify target transcripts. In a previous study, alternative splicing (AS) of *CSD1* in peanut (*Arachis hypogaea* L.) resulted in a positive correlation between miR398 and enhanced drought tolerance [21], which may be the reason why the *SOD* variants generated by alternative splicing might be associated with the spatial-temporal regulation of gene expression. Remarkably, the AS of *GmCSD1a-1* and *GmCSD1b-1*, which are both lack of gma-miR398c binding sites, were not detected by 5′ RACE in our data, while the expression levels of *GmCSD1a* and *GmCSD1b* genes were still negatively correlated with gma-miR398c (Figure S2B). Therefore, evaluation of individual transcripts from among the present transcriptome data might contribute to elucidation of the drought-responsive mechanism of miRNA-target regulation in soybean. Notably, only *CSD1* in common bean (*Phaseolus vulgaris* L.) and barley was separately identified using 5′ RACE, which failed to detect other target genes [34, 35]. In our study, we proved that gma-miR398c is capable of interacting with *GmCSD1a*/*b*, *GmCSD2a/b/c*, and *GmCCS*, probably because of their relatively low expression levels in that tissue or translation repression.

Meanwhile, plant CSDs play an important role in antioxidant systems by scavenging O_2_^.−^, which is crucial to minimize cell damage caused by excessive accumulation of ROS [24, 25]. Moreover, the activation of different CSDs in Arabidopsis shows varying dependence on CCS [36]. However, the genes encoding SOD-related isoforms in soybean are unwell explored and poorly known [37]. Wang et al. [38] reported that *GmCZ-SOD1* (Glyma05g04170) may be important in the adaptive evolution of nitrogen limitation in soybean, whereas it was identified as *GmCCS* (Glyma.05 g055000) encoding a copper chaperone for superoxide dismutase in phylogenetic analysis (Figure S5A). We determined that GmCCS is expressed predominantly in chloroplasts (Fig. 4), similar to homologous genes in Arabidopsis [39]. These results might be valuable for further understanding of the redox status under drought stress. Overall, our above data may contribute to claim the evidence of the characteristics of the gma-miR398c regulatory network and to aid in elucidation of drought tolerance mechanisms in soybean.

### Drought-responsive gma-miR398c negatively regulated drought tolerance in transgenic plants

In previous studies, overexpressing miR398 negatively regulates plant stress tolerance in *Arabidopsis thaliana* and *Nicotiana benthamiana* [12, 13]. The *Mla* and *Rom1* loci decrease miR398 expression and increase HvSOD1 accumulation, which confers powdery mildew resistance in barley (*Hordeum vulgare* L.) [35]. Loss-of-function mutants of miR398 and overexpression of the miR398-resistant *CSD1/2* and *CCS* indicated that the miR398 regulatory network confers stress tolerance in plants [40, 41]. Recently, overexpression of osa-miR398b and mutants in *CSD1* and *CSD2* positively regulated plants stress tolerance by altering expression of multiple SODs, indicating that in different plant species miR398 might play different roles in stress resistance [14, 15]. However, there was no direct genetic evidence neither a thorough phenotypic analysis that indicated soybean miR398 was involved in drought resistance. In the current study, gma-MIR398c expression was strongly inhibited in response to drought stress in soybean. Thus, we observed that OE-miR398c Arabidopsis showed significantly inhibited germination and survival frequencies (Fig. 5), and the rate of water loss of detached leaves was increased compared with that of WT and OE-vector plants (Fig. 6c), which indicated that gma-miR398c may strongly reduce plant drought resistance. Actually, we also analysed the phenotypes of transgenic lines overexpressing other members of the gma-miR398 family but did not observe any differences (data not shown). However, we do not exclude the possibility that other gma-miR398s might also regulate plant stress resistance.

Notably, overexpression of common bean pvu-miR398a, a dicistronic unit co-transcribed by pvu-miR398a and pvu-miR2119, using a common beans transgenic hairy-root system reduced plant stress tolerance by mediating *CSD1*, and was predicted to regulate the stress resistance of legumes [34]. In soybean, a dicistronic unit co-transcribed by gma-miR398a and gma-miR2119 was also identified, but it showed a low expression level in roots and leaves (Fig. 2c), and overexpression of gma-miR398a did not reduce Arabidopsis drought tolerance. Collectively, these results imply that different miR398 family members may have diverse effects in legumes [9]. Therefore, overexpression and knockout assays of gma-miR398c were performed using a soybean hairy-root transformation system. Here, we observed that gma-miR398c expression levels were decreased and *GmCSD1a/b, GmCSD2a/b/c* and *GmCCS* genes were elevated in KO-1 and KO-2 plants (Fig. 8b, c), similar to findings reported for Arabidopsis miR398 mutants [40]. Recently, mutations in *CSD1* and *CSD2* led to conferred rice resistance, whereas the *CCS* mutant displayed lower CSD activity and enhanced susceptibility [14]. In our study, the relative electrolyte leakage, O_2_^.−^ content and stomatal aperture were significantly increased in OE-miR398c plants under simulated drought stress, resulting in reduced drought tolerance (Fig. 7, Fig. 8a). However, releasing multiple *GmCSD1a/b, GmCSD2a/b/c* and *GmCCS* genes from repression by CRISPR/Cas9 editing of miR398c was helpful to scavenge O_2_^.−^ and contributed to increased drought resistance (Fig. 7, Fig. 8). Taken together, the present analyses indicated that miR398c in soybean is a strongly negative regulator that reduces plant drought tolerance. Therefore, knockout of gma-miR398c will be sufficient in molecular breeding to achieve crop improvement.

## Conclusion

Correlation analysis of sRNA-seq, degradome-seq, and mRNA-seq revealed a significant induction of gma-miR398c as a major gene negatively regulates multiple peroxisome-related genes (*GmCSD1a/b, GmCSD2a/b/c* and *GmCCS*) in soybean under drought stress conditions. Furthermore, functional investigation confirmed that drought-responsive gma-miR398c could negatively regulate soybean drought tolerance via *GmCSDs* and *GmCCS*, not affected by alternative splicing (AS) of *CSD1*. The knock out of gma-miR398c will enhance our knowledge in functional validation of gma-miR398c and will be valuable in molecular breeding to improve the drought tolerance of cultivated soybean.

## Methods

### Soybean seedling growth conditions and simulated drought stress treatment

The cultivated soybean seeds of *Glycine max* (Williams 82) provided by Northeast Institute of Geography and Agroecology, Chinese Academy of Sciences were used for our study. The seeds were surface-sterilized in ethanol for at least 10 min and then washed several times with distilled water. An equal number of seeds were added to pots containing half-strength (1/2) Hoagland’s nutrient solution and incubated at 25 °C/18 °C (day/night) with a 16-h/8-h (light/dark) photoperiod under photosynthetic photon flux density of 80 μmol m^− 2^ s^− 1^. Once the first compound leaf had expanded, three replicates of each group were exposed to 7, 8%, or 9% polyethylene glycol (PEG) 8000 for 1, 3, 6, 9, 12, or 24 h to simulate drought. An untreated (0 h) pot was used as a control. Physiological indices were measured using superoxide dismutase (SOD), malondialdehyde (MDA), and catalase (CAT) detection kits (A001–1, A003–1, and A007–1, Nanjing Jiancheng Bioengineering Institute, Nanjing, China). Notice that, the phenotype showed that the leaves of seedlings treated with 9% PEG 8000 were wilted substantially, and the SOD activity and contents of MDA and H_2_O_2_ were significantly affected at 6 and 12 h (Figure S6). Therefore, all samples of three replicates treated with 8% PEG 8000 at 0, 6, and 12 h were used to prepare RNA libraries.

### Total RNA extraction and mRNA, degradome and sRNA library preparation

The RNA sequencing (RNA-seq) experiment was conducted using three replicates. Leaf and root samples were collected from seedlings treated with 8% PEG 8000 for different durations. All samples were immediately frozen in liquid nitrogen and stored at − 80 °C until analyzed. Total RNA was extracted using the TRIzol® Reagent (Invitrogen, Carlsbad, CA, USA). The concentration, purity, and integrity of the total RNA were analyzed using a NanoPhotometer® spectrophotometer (Implen, Westlake Village, CA, USA), the Qubit™ RNA Assay Kit with a Qubit 2.0 Fluorometer (Life Technologies, Camarillo, CA, USA), and the RNA Nano 6000 Assay Kit with the Agilent Bioanalyzer 2100 system (Agilent Technologies, Santa Clara, CA, USA).

### Transcriptome library construction and sequencing

The mRNA-seq experiment was completed using three replicates of 18 samples for the leaf and root samples collected at 0, 6, and 12 h. The mRNA-seq libraries were prepared from 1 μg RNA per sample. The libraries were sequenced using the HiSeq X™ Ten platform. The paired-end reads were filtered by removing the adapters and low-quality sequences. The remaining high-quality reads were mapped to the Glycine_max.Wm82.a2.v1 reference genome (http://phytozome.jgi.doe.gov/pz/portal.html). The mapped reads were further analyzed using the TopHat2 software. First, the mapped reads were spliced using the Cufflinks program and new genes were detected by filtering out sequences that encoded short peptide chains (fewer than 50 amino acids) or contained only one exon. Furthermore, the sequences were compared with those of the non-redundant (nr), Swiss-Prot, Clusters of Orthologous Groups of proteins (COG), Clusters of Protein homology (KOG), and Kyoto Encyclopedia of Genes and Genomes (KEGG) databases using the BLASTX online tool. The GO annotations of genes assigned by the Blast2GO software were based on the results of the comparison with nr database protein sequences. KEGG Orthology information for the genes was obtained using KOBAS 2.0 [42]. The amino acid sequences encoded by the genes were compared with protein sequences in the Pfam database using the HMMER program for the annotations [43]. The mRNA levels were normalized based on the fragments per kilobase of transcript per million mapped reads (FPKM) value. The differentially expressed genes (DEGs) were detected based on the following parameters: |log_2_FC| ≥ 1 with FDR < 0.05 and CPM > 1.

### Small RNA sequencing and miRNA identification

For the sRNA-seq experiment, mRNA libraries were prepared for the leaf and root tissues collected at 0, 6, and 12 h. Purified RNA (1.5 μg) was used to construct a sequencing library with the NEBNext® Ultra Small RNA Sample Library Prep Kit for Illumina® (New England Biolabs, Ipswich, MA, USA). The libraries were sequenced using the Illumina HiSeq 2500 platform. The resulting paired-end reads were filtered by removing contaminants and low-quality reads, and reads containing poly-Ns and adapters. In addition, sequences shorter than 18 nucleotides or longer than 30 nucleotides were eliminated to ensure the accuracy of all downstream analyses. Using the Bowtie software, the clean reads were mapped based on sequence alignments using the SILVA, GtRNAdb, Rfam, and Repbase databases to filter out ribosomal RNA, transfer RNA, small nuclear RNA, small nucleolar RNA, and other non-coding RNA and repeats. The remaining reads were used to detect known miRNAs based on comparisons with miRNAs in the miRBase database (version 21.0) (http://www.mirbase.org/). The miRDeep-p tool was applied to predict plant novel miRNAs [44]. Potential precursor sequences were obtained by analysing the genomic position information. Based on the distribution of the miRNA characteristics (i.e., mature, star, and loop regions) as well as precursor energy information obtained with the RNAfold and randfold tools [45], we scored the newly identified miRNAs using a Bayesian model. The frequency of the miRNAs in all libraries was expressed as transcripts per million (TPM). The differentially expressed miRNAs (DEMs) were detected based on the following parameters: |log_2_FC (fold change)| ≥ 1 with false discovery rate (FDR) < 0.05 and counts per million (CPM) > 1.

### Degradome sequencing, target identification, and data analysis

The leaf and root samples collected at different time points were respectively mixed to obtain two pools for degradome sequencing. The degradome libraries were prepared using approximately 25 μg total RNA from the mixed leaf and mixed root samples [46]. Biotinylated random primers were annealed to poly-A^+^ RNA, which was used as the template. The RNA fragments with the biotin tag were obtained using streptavidin. Only RNAs with 5′ monophosphates were ligated with a 5′ adapter. The resulting RNA fragments were used as templates for first-strand cDNA synthesis. In addition, sequencing primers were designed consistent with the 5′ adapter sequence, which corresponded to the first 50 nucleotides from the 5′ site of the cleaved RNAs. Sequencing (50 bp single-end reads) was performed using the HiSeq 2500 platform. The spliced miRNA targets were identified and classified using the CleaveLand 3.0 pipeline. Putatively identified transcripts were divided into five categories: category 0: more than one raw read at the cleaved site, abundance at the site is equal to the only maximum on the transcript; category 1: more than one raw read at the cleaved site, abundance at the site is equal to a maximum on the transcript and there is more than one maximum; category 2: more than one raw read at the cleaved site, abundance at the site is less than the maximum but more than the median value for the transcript; category 3: more than one raw read at the cleaved site, abundance at the site is less than or equal to the median value for the transcript; and category 4: only one raw read at the cleaved site [47]. The potential target genes were subjected to gene ontology (GO) analysis using the AgriGO toolkit [48].

### Verification by RT-qPCR analysis

The total RNA extracted from plants treated with 8% PEG 8000 (0, 6, and 12 h) was used for quantitative reverse transcription PCR (RT-qPCR) and stem-loop RT-qPCR analyses of genes (mRNA) and miRNAs, respectively, in accordance with the MIQE standards [49]. All primer sequences were designed and synthesized based on the miRNA and mRNA sequences (Table S7) (Genewiz, Beijing, China). For the RT-qPCR analysis of genes, total RNA samples were treated with RNase-free DNase I to eliminate any contaminating genomic DNA. The RNA was then used as the template for cDNA synthesis with the PrimeScript™ RT Reagent Kit with gDNA Eraser (Takara). The stem-loop RT-qPCR analysis of miRNA involved a special step during which 1 μg RNA was reverse-transcribed with PrimeScript RT Enzyme Mix I and miRNA-specific stem-loop primers. The RT-qPCR and stem-loop RT-qPCR were completed using the SYBR® Premix Ex Taq™ II kit (Takara). We selected *GmActin* and *GmEF1b* for RT-qPCR, and gma-miR1520d for stem-loop RT-qPCR as internal controls. The RT-qPCR and stem-loop RT-qPCR experiments were completed with three technical replicates for each biological replicate. Relative expression levels were quantified using the 2^−ΔΔCt^ method.

### Analysis of the interaction between gma-miR398 and their targets

A modified RNA ligase-mediated 5′ rapid amplification of cDNA ends (RACE) completed with the GeneRacer™ Kit (Invitrogen) was used to examine RNA cleavage*.* First, the RNA oligo was ligated to 3 μg total RNA from each mixed leaf and mixed root sample. Using random primers, we generated cDNA from the mRNA with the 5′ RACE adapter by reverse transcription. The GSP and GSP-Nested primers from the kit were used to amplify the 5′ end of the cleaved transcript with two gene-specific primers by nested PCR and touchdown PCR. Finally, the nested PCR product was incorporated into the pEASY-T1 vector (Table S7) (TransGen, Beijing, China), inserted into *Escherichia coli* (DH5α) cells and sequenced.

The Wild-type *Arabidopsis thaliana* (ecotype Columbia) from Northeast Institute of Geography and Agroecology, Chinese Academy of Sciences was used for all Arabidopsis experiments. Arabidopsis mesophyll protoplasts were transiently transformed to clarify the interactions between miR398 and their targets [6]. We synthesized four interaction sequences (i.e., for *GmCSD1a*, *GmCSD1b*, *GmCSD2a/b/c*, and *GmCCS*) and four synonymous mutation sequences and inserted them into the HBT-sGFP(S65T)-NOS vector (Figure S7). Mesophyll protoplasts were extracted from 4-week-old Arabidopsis, wild-type Arabidopsis (WT), empty vector overexpression plants (OE-vector), and gma-miR398c overexpression plants (OE-miR398c). Following DNA-PEG-calcium–mediated transformation [50], the green fluorescent protein (GFP) and chloroplast autofluorescence signals were observed using an IX51 inverted fluorescence and phase contrast microscope (Olympus, Japan).

### Subcellular localization of GmCSDs and GmCCS

Firstly, we constructed a phylogenetic tree consisting of *GmSOD*-related genes to characterize potential members, and 14 distinct open reading frames potentially encoding 13 *GmSOD* genes and one *GmCCS* gene were detected in soybean. These genes contribute to the degradation of reactive oxygen species (ROS) (Figure S5). To determine the subcellular localization of GmCSDs and GmCCS via transient expression in tobacco leaves and Arabidopsis mesophyll protoplasts, the coding regions were cloned into the pCAMBIA-1302 vector and HBT-sGFP(S65T)-NOS vector for fusion with GFP, respectively (Table S7). The *Agrobacterium tumefaciens* strain EH105 harboring 35S:GmCSDs-GFP, 35S:GmCCS-GFP, or 35S:GFP was separately transformed into tobacco leaves, and a nucleus marker (NLS) [51] and peroxisome marker (SKL) [52] were used to co-localize with functional genes. After 48 h, leaf epidermal cells from transformed tobacco were observed with a confocal microscope (C2-ER, Nikon, Japan). Fluorescence of GFP, chloroplasts, and the markers was stimulated at 488, 640, and 561 nm, respectively.

### Generation of transgenic *Arabidopsis thaliana* and simulated drought stress treatment

The gma-miR398c precursor sequence (gma-MIR398c) was inserted in the pCAMBIA-3301 vector using the *Bgl*II and *Pml*I sites between the CaMV-35S promoter and Nos-terminator. Wild-type Arabidopsis was then transformed with either the empty vector or the vector carrying gma-MIR398c using the EHA105 strain *Agrobacterium tumefaciens* floral-dip method. Seeds from T_2_ transgenic lines exhibiting a segregation ratio of 3:1 and from three homozygous T_3_ lines with the highest transgene expression levels were screened on Murashige and Skoog (MS) medium supplemented with BASTA (10 mg l^− 1^ active ingredient).

Arabidopsis seeds were surface-sterilized and imbibed at 4 °C for 48 h in darkness. The seeds were then sown on MS medium supplemented with 3% sucrose (w/v), 0.8% agar (w/v), and 250 mM or 300 mM D-mannitol to induce low-water-potential stress. The germination frequency (%) was calculated after treatment for 14 days, and survival rate of Arabidopsis seedlings after 10 days cultivation on 300 mM D-mannitol medium. The plates were incubated at 22 °C under a 16-h/8-h (light/dark) photoperiod. For simulated drought treatment of soil-grown plants, an equal number (25:25) of transgenic and WT Arabidopsis plants were cultivated side-by-side in the same container containing soil:vermiculite (7:3) in a greenhouse at 22 °C under a 12-h/12-h (light/dark) photoperiod with 50% relative humidity. Importantly, water was withheld from 3-week-old Arabidopsis plants until they wilted (i.e., for at least 3 weeks). The watering of seedlings was resumed and plants were analyzed after 3 days. Water loss was measured using detached leaves collected from 4-week-old Arabidopsis plants. The detached leaves were weighed at 1-h intervals at room temperature (22 °C) under normal light with 70% relative humidity. The water loss rate was calculated based on the percentage of the initial fresh weight that remained at different time points. The germination, survival, and water loss rates for five independent replicates of each transgenic line were estimated.

### *Agrobacterium Rhizogenes*-mediated soybean hairy roots transformation and drought stress

The CRISPR/Cas9 vector was obtained from Biogle (Cat#BGK041, Hangzhou, China). Using the web-based tool CRISPR-P (http://crispr.hzau.edu.cn/), sgRNA-1 and sgRNA-2 sequences were designed on the hairpin and mature sequences of gma-MIR398c, respectively. The sequences of sgRNA-1 and sgRNA-2 with their specific primers were cloned into the CRISPR/Cas9 vector including the soybean U6-specific promoter (pCas9-U6-sgRNA) as a knockout vector (KO-1 and KO-2) (Table S7). The pCAMBIA3301-miR398c vector was used as an overexpression vector. The hypocotyls of 5-day-old soybean seedlings were injected with *Agrobacterium rhizogenes* (K599) harboring the pCas9-U6-sgRNAs or pCAMBIA3301-miR398c plasmid [53]. The seedlings were cultivated in humidity for 6 days, after which the top leaves were removed and the wound sites were covered with vermiculite for about 15 days until hairy roots about 10 cm in length had developed (Figure S8). The seedlings were transferred to 1/2 Hoagland’s nutrient solution for recovery after the original roots were excised. Seedlings of uniform growth with hairy roots were subjected to 6% PEG 8000 for 48 h to induce simulated drought stress. A total of ten independent replicates were performed.

### CRISPR/Cas9 activity assessment

To detect the capacity and efficiency of CRISPR/Cas9 for editing the precursor sequence of gma-miR398c, sgRNA-1 and sgRNA-2 of different regions were inserted in the CRISPR/Cas9 vector (Figure S9A). PCR primers for genomic DNA from transgenic roots were designed to amplify about 705 bp containing the target site (Table S7). The PCR products were denatured and renatured to produce heteroduplexes. Given the absence of an appropriate restriction enzyme site in gma-MIR398c, the T7E1 (E001L, ViewSolid, Beijing, China) assay was performed to detect CRISPR/Cas9-induced mutations. The mutant sites were identified by subcloning and sequencing. To calculate insertion/deletion (indel) frequencies in accordance with the formula: indel (%) = 100 × *a* / (*a* + *b* + *c*), where *a* is the intensity of the undigested band, and *b* and *c* are the intensities of the other bands, we measured the gel band intensities with ImageJ [54]. We detected the capacity and efficiency of CRISPR/Cas9 for editing gma-MIR398c and identified mutants in 9 and 8 out of 18 independent transgenic hairy roots with indel frequencies ranging from 3.9 to 19.3% and from 2.9 to 10.3%, respectively (Figure S9B). To subclone and sequence the positive PCR products, the indels of mutants at the target sites were conformed (Figure S9C).

### Relative electrolyte leakage and superoxide free radical (O_2_^.−^) assay

After stress treatment for 48 h, the second trifoliate leaf from the shoot apex of control and treated seedlings was used for a relative electrolyte leakage assay. The leaves of 10 seedlings for each treatment were vacuum-infiltrated for 45 min in water, and placed for 1 h at 25 °C. The conductivity (R1) was measured. The leaves were then autoclaved for 15 min and shaken until water dropped to 25 °C. Relative electrolyte leakage (%) was calculated as the ratio R1/R2. The production of O_2_^.−^ was analyzed following the method of Elstner and Heupel [55].

### Stomatal aperture measurement

The composite plants were treated with 6% PEG 8000 for 3 h, and plants were placed in 1/2 Hoagland’s nutrient solution as a control. The stoma of the second trifoliate leaf from the shoot apex of control and treated plants was observed with a Leica TCS-SPE confocal microscope.

### Data analysis

Data were subjected to analysis of variance. Student’s *t*-test was used to assess the significance of differences between the means of two datasets (***P < 0.001, **P < 0.01 and *P < 0.05). The data are presented herein as the mean ± standard deviation.

### Online data deposition

The sRNA-seq, degradome-seq, and mRNA-seq raw data have been deposited in the NCBI database SRA (PRJNA407016) and the SRR accession numbers are SRR6122987 to SRR6123012.

## Supplementary information

**Additional file 1: Figure S1.** Target plots (t-plots) of identified gma-miR398 targets. (A) The cleaved site of *GmCCS;* (B) The cleaved site of *GmCSD1a;* (C) The cleaved site of *GmCSD1b;* (D) The cleaved site of *GmCSD2a;* (E) The cleaved site of *GmCSD2b.* The X axis indicated the site position of target cDNA, the Y axis indicated the normal abundance of raw tags. The red colored line on the target transcript indicated the cleavage site.

**Additional file 2: Figure S2.** Effects of alternative splicing from *GmCSD1a/b* for miR398. (A) The sequence alignment of ath-miR398s and gma-miR398s; (B) The information of alternative splicing from *GmCSD1a/b.* The red colored region on the target transcripts indicated all transcripts of *GmCSD1a* and *GmCSD1b*, except for glyma.03 g242900.1 and glyma.19 g240400.1, contains the cleavage site at the 5′ UTR region.

**Additional file 3: Figure S3.** Details regarding the validation of the gma-miR398s cleavage sites in the transcripts of *GmCSD1a*, *GmCSD1b*, *GmCCS* and *GmCSD2a* analyzed by 5′ RACE. The red colored arrow on the target transcript indicated the cleavage site and the number next to the arrow in the alignment between the miRNA and the target was the cDNA position corresponds to the detected cleavage site.

**Additional file 4: Figure S4.** Subcellular localization of GmCSDs and GmCCS genes in *Arabidopsis* mesophyll protoplasts. 35S:GmCSDs-GFP, 35S:GmCCS-GFP, or 35S:GFP was separately transformed into Arabidopsis mesophyll protoplasts. The green fluorescence signals were obtained by an IX51 inverted fluorescence and phase contrast microscope. Scale bars = 50 μm.

**Additional file 5: Figure S5.** Phylogenetic tree of SOD-related genes and an analysis of the reactive oxygen species degradation pathway. (A) The maximum likelihood tree was constructed using the MEGA 6.0 program and was based on the full-length amino acid sequences encoded by the SOD-related genes, which were named according to their names in Arabidopsis database. Bootstrap = 1000. (B) Examination of the SOD-related genes (*GmSODs*, *GmGPXs* and *GmCATs*) involved in the degradation of reactive oxygen species based on soybean transcriptome data analyzed using Phytozome. The original expression values underwent a Z-score normalization; normalized signal values = log_10_(FPKM).

**Additional file 6: Figure S6.** Response of soybean seedlings to different PEG concentrations. (A) Performance of soybean seedlings under different stresses for 12 h. (B) SOD contents of seedlings under 8% PEG stress for different durations. (C) MDA contents of seedlings under 8% PEG stress for different durations. (D) H_2_O_2_ contents of seedlings under 8% PEG stress for different durations In all panels, values are average of three biological replicates ± SD, different letters and asterisks indicate significant difference applying ANOVA (*, *P <* 0.05; **, *P <* 0.01; ***, *P <* 0.001).

**Additional file 7: Figure S7.** Map of the vector constructed to validate the interaction between gma-miR398c and its target genes in transiently transformed Arabidopsis mesophyll cells. (A) The HBT-sGFP(S65T)-NOS vector was used for vector construction. (B) The 21 bp conserved sequence of cleavage site was inserted into HBT-sGFP(S65T)-NOS vector to get the interaction vectors which called as HBT-sGFP(S65T)-NOS-Target.

**Additional file 8: Figure S8.** Different stages of the soybean hairy root transformation. (a) The 6-day-old seedlings of soybean. (b) Inoculation with bacterial paste. (c) Stabbing of the hypocotyl close to the cotyledonary node. (d) Growth of soybeans in pots. (e) Place a small bowl of water for 5 to 6 days to keep water. (f) Hairy roots after (e) step. (g) The wounding sites of soybean are cultivated by wet vermiculite. Soybean cultivated for about 20 days after inoculation. (h) The growth of hairy roots after the removal of the primary root. (i) Soybean hairy root GUS staining.

**Additional file 9: Figure S9.** The editing system of gma-miR398c gene in soybean hairy root by CRISPR/Cas9. (A) Soybean precursor miR398c stem-loop structure and sgRNA information. (B) T7E1 enzyme digestion sgRNA-1 and sgRNA-2 editing efficiency. Lanes WT and WT*, undigested and digested wild-type controls, respectively. The red arrowhead indicates the digested bands. The numbers at the bottom of the gels indicate mutation frequencies measured according to band intensities. M, DL2000 ladder DNA marker. (C) Cloning and sequencing of the digested bands. sgRNA-1 and sgRNA-2 target site editing information.

**Additional file 10: Table S1.** Summary of sRNA, degradome and mRNA sequencing for individual libraries.

**Additional file 11: Table S2.** Known and novel miRNA identified in each soybean sRNA-seq libraries.

**Additional file 12: Table S3.** Differentially expressed miRNAs for different treatment durations in leaves and roots.

**Additional file 13: Table S4.** Details of miRNA targets identified by degradome sequencing

**Additional file 14: Table S5.** Differentially expressed genes for different treatment durations.

**Additional file 15: Table S6.** Information of all known and novel genes identified in soybean.

**Additional file 16: Table S7.** Details regarding the primers used in this study.

## Data Availability

Our data sets supporting the results of this study are included within the article and its additional files. Sequencing data used in this manuscript can be found in the NCBI database SRA (PRJNA407016) and the SRR accession numbers are SRR6122987 to SRR6123012.

## Abbreviations

Gma-miR398a/b/c/d: *Glycine max* mature gma-miR398s
Gma-MIR398s: *Glycine max* gma-miR398 precursors
GmCSDs: Cu/Zn superoxide dismutase genes
GmCCS: Copper Chaperone for superoxide dismutase genes
5′-RACE: RNA ligase-mediated rapid amplification of 5′-cDNA ends
RT-qPCR: Reverse transcription quantitative real-time PCR
Vector: Empty plant expression vector
OE: Over-expression transgenic plants
WT: Wild-type plants
