# Supplementary figures and images for: Integration of sRNA, degradome, transcriptome analysis and functional investigation reveals gma-miR398c negatively regulates drought tolerance via GmCSDs and GmCCS in transgenic Arabidopsis and soybean
Source: BMC Plant Biol. 2020 May 5;20:190. doi: 10.1186/s12870-020-02370-y (PMC7201782; doi:10.1186/s12870-020-02370-y)

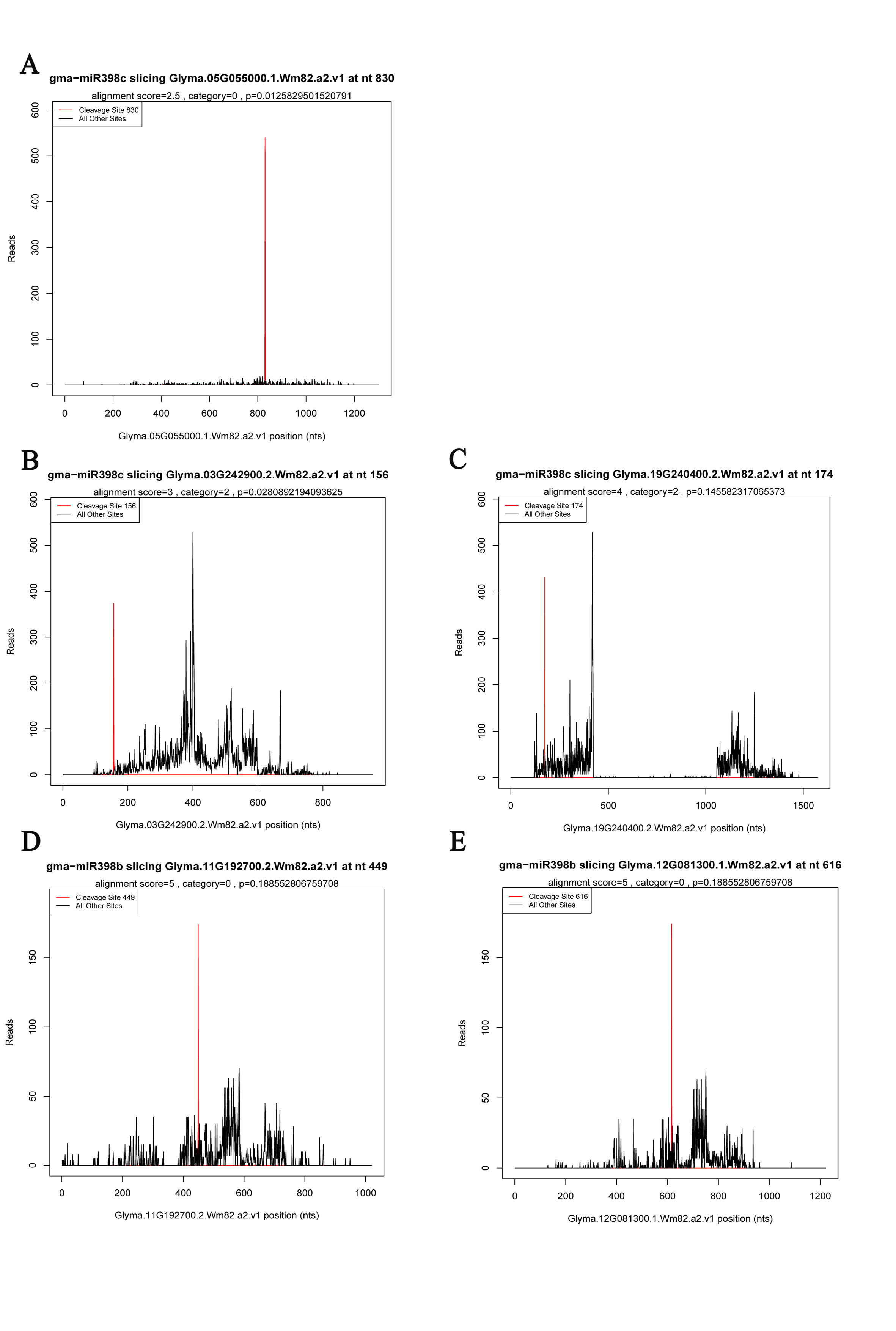

Supplement: Supplementary file 1 — Additional file 1: Figure S1. Target plots (t-plots) of identified gma-miR398 targets. (A) The cleaved site of GmCCS; (B) The cleaved site of GmCSD1a; (C) The cleaved site of GmCSD1b; (D) The cleaved site of GmCSD2a; (E) The cleaved site of GmCSD2b. The X axis indicated the site position of target cDNA, the Y axis indicated the normal abundance of raw tags. The red colored line on the target transcript indicated the cleavage site. [file 12870_2020_2370_MOESM1_ESM.jpg]

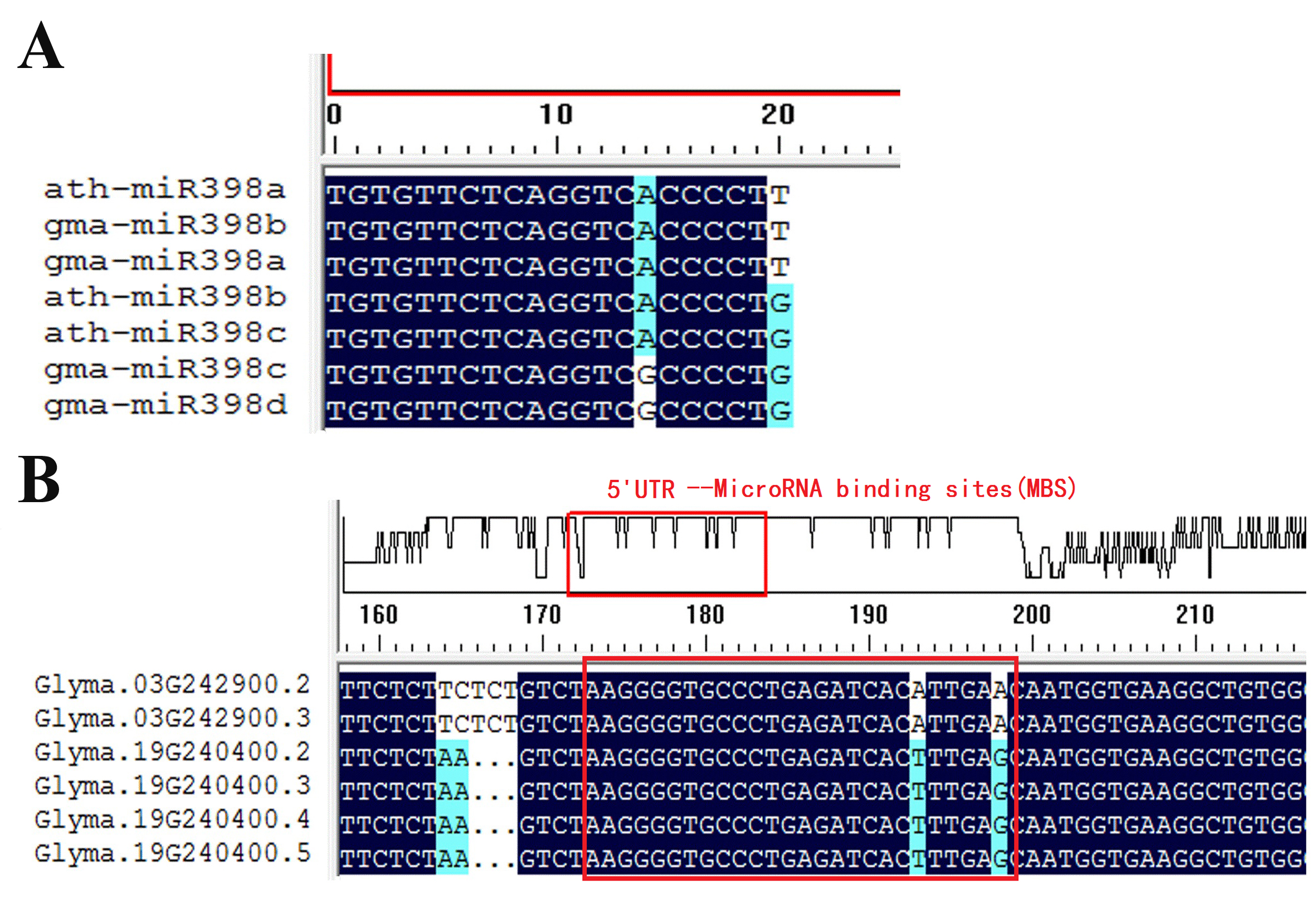

Supplement: Supplementary file 2 — Additional file 2: Figure S2. Effects of alternative splicing from GmCSD1a/b for miR398. (A) The sequence alignment of ath-miR398s and gma-miR398s; (B) The information of alternative splicing from GmCSD1a/b. The red colored region on the target transcripts indicated all transcripts of GmCSD1a and GmCSD1b, except for glyma.03 g242900.1 and glyma.19 g240400.1, contains the cleavage site at the 5′ UTR region. [file 12870_2020_2370_MOESM2_ESM.jpg]

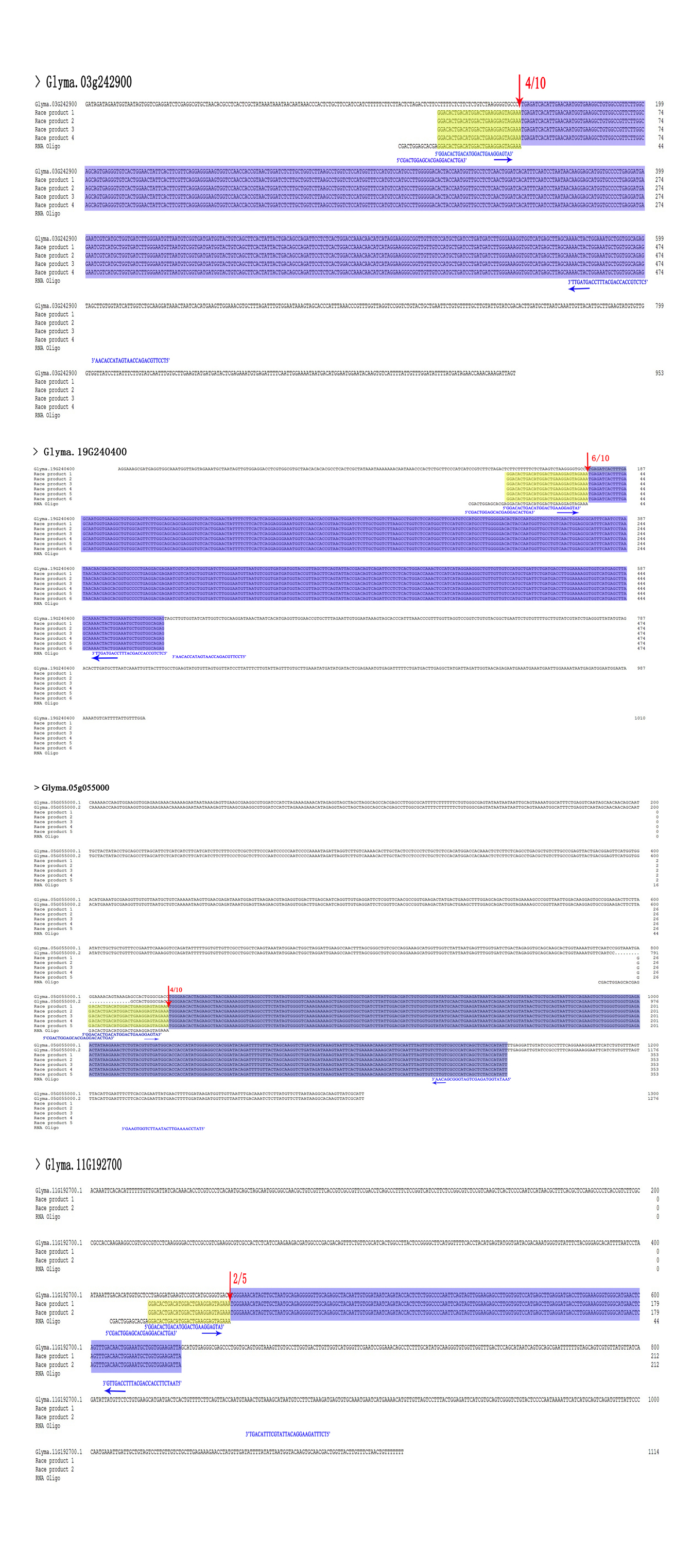

Supplement: Supplementary file 3 — Additional file 3: Figure S3. Details regarding the validation of the gma-miR398s cleavage sites in the transcripts of GmCSD1a, GmCSD1b, GmCCS and GmCSD2a analyzed by 5′ RACE. The red colored arrow on the target transcript indicated the cleavage site and the number next to the arrow in the alignment between the miRNA and the target was the cDNA position corresponds to the detected cleavage site. [file 12870_2020_2370_MOESM3_ESM.jpg]

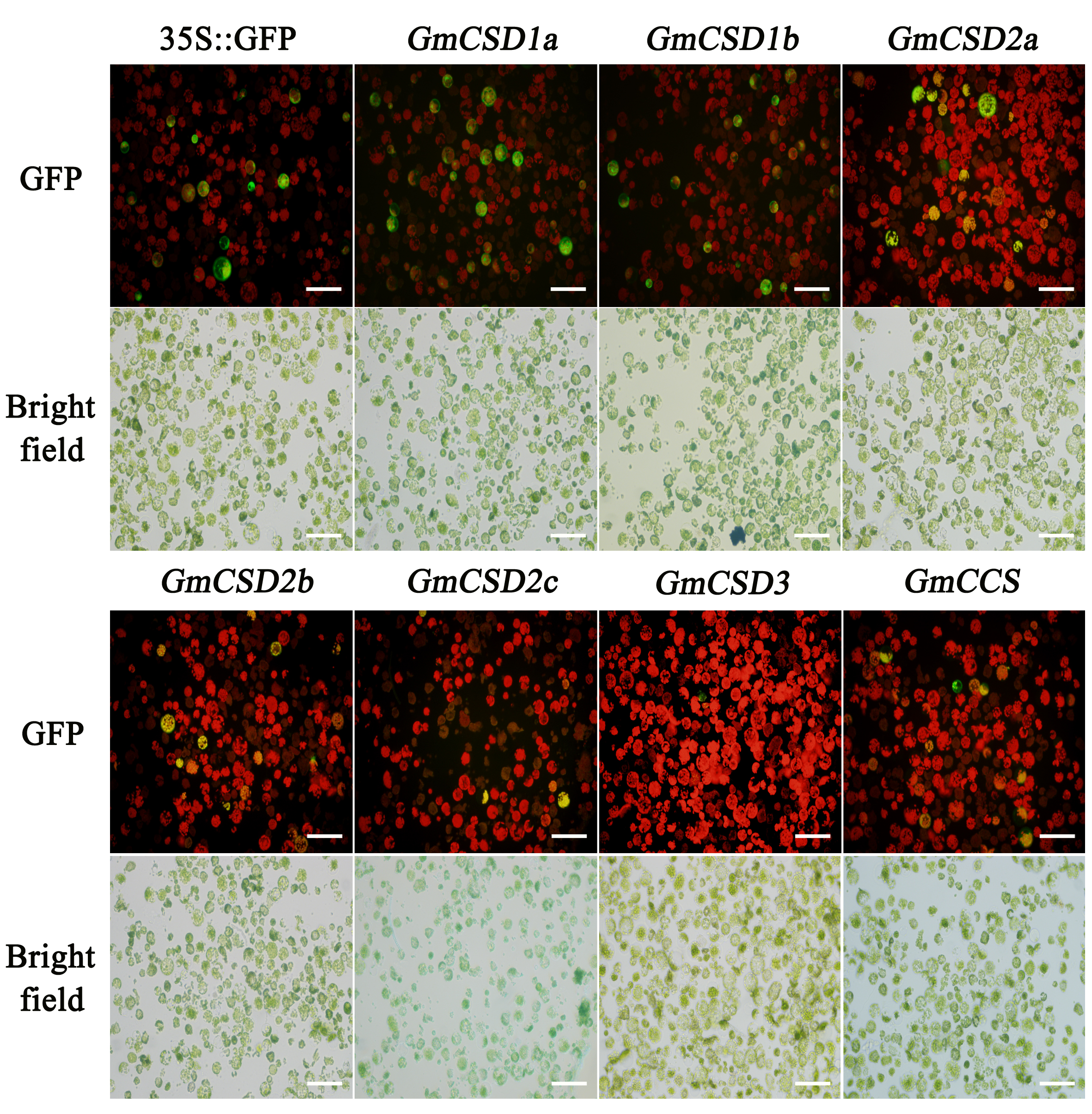

Supplement: Supplementary file 4 — Additional file 4: Figure S4. Subcellular localization of GmCSDs and GmCCS genes in Arabidopsis mesophyll protoplasts. 35S:GmCSDs-GFP, 35S:GmCCS-GFP, or 35S:GFP was separately transformed into Arabidopsis mesophyll protoplasts. The green fluorescence signals were obtained by an IX51 inverted fluorescence and phase contrast microscope. Scale bars = 50 μm. [file 12870_2020_2370_MOESM4_ESM.jpg]

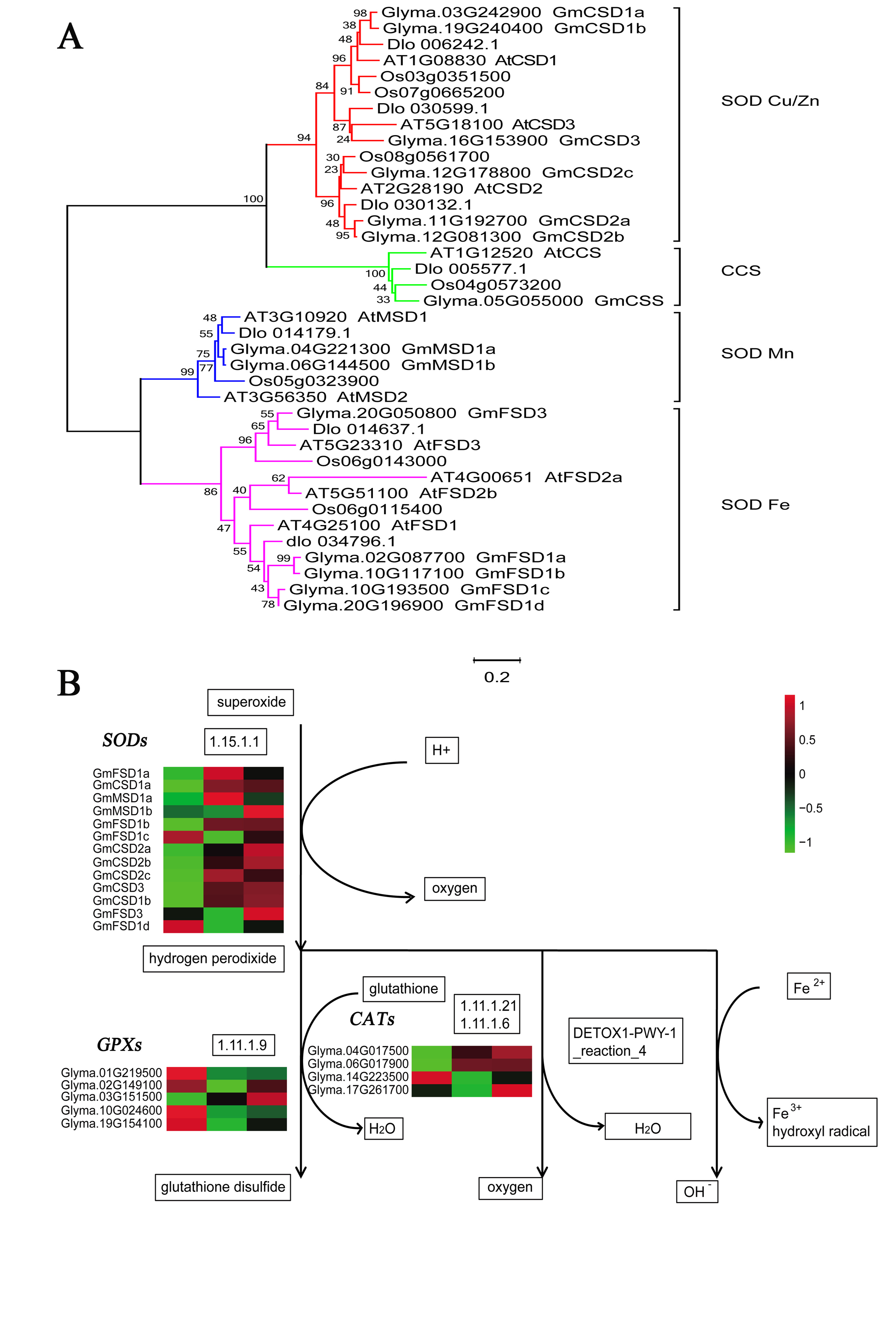

Supplement: Supplementary file 5 — Additional file 5: Figure S5. Phylogenetic tree of SOD-related genes and an analysis of the reactive oxygen species degradation pathway. (A) The maximum likelihood tree was constructed using the MEGA 6.0 program and was based on the full-length amino acid sequences encoded by the SOD-related genes, which were named according to their names in Arabidopsis database. Bootstrap = 1000. (B) Examination of the SOD-related genes (GmSODs, GmGPXs and GmCATs) involved in the degradation of reactive oxygen species based on soybean transcriptome data analyzed using Phytozome. The original expression values underwent a Z-score normalization; normalized signal values = log10(FPKM). [file 12870_2020_2370_MOESM5_ESM.jpg]

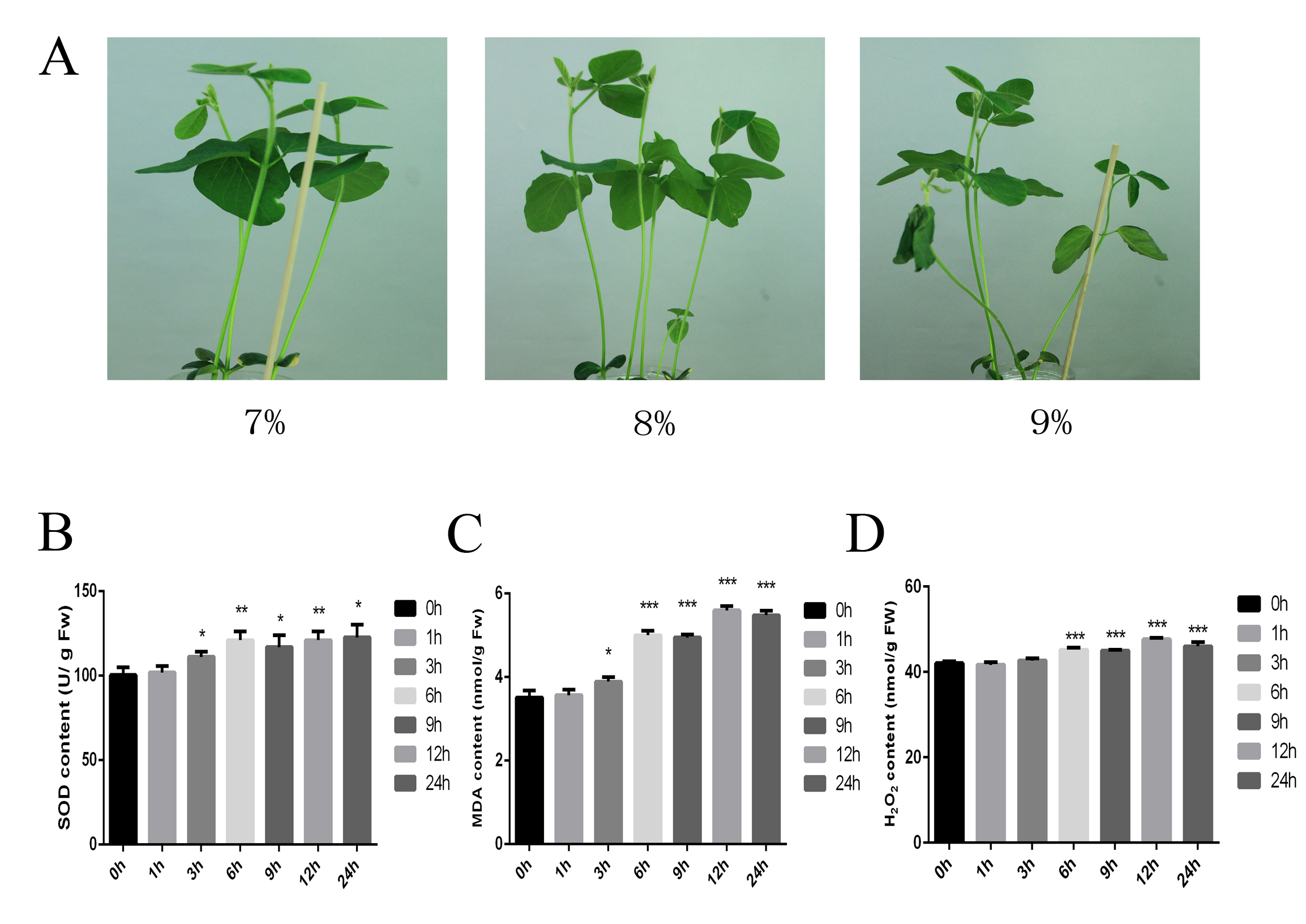

Supplement: Supplementary file 6 — Additional file 6: Figure S6. Response of soybean seedlings to different PEG concentrations. (A) Performance of soybean seedlings under different stresses for 12 h. (B) SOD contents of seedlings under 8% PEG stress for different durations. (C) MDA contents of seedlings under 8% PEG stress for different durations. (D) H2O2 contents of seedlings under 8% PEG stress for different durations In all panels, values are average of three biological replicates ± SD, different letters and asterisks indicate significant difference applying ANOVA (*, P < 0.05; **, P < 0.01; ***, P < 0.001). [file 12870_2020_2370_MOESM6_ESM.jpg]

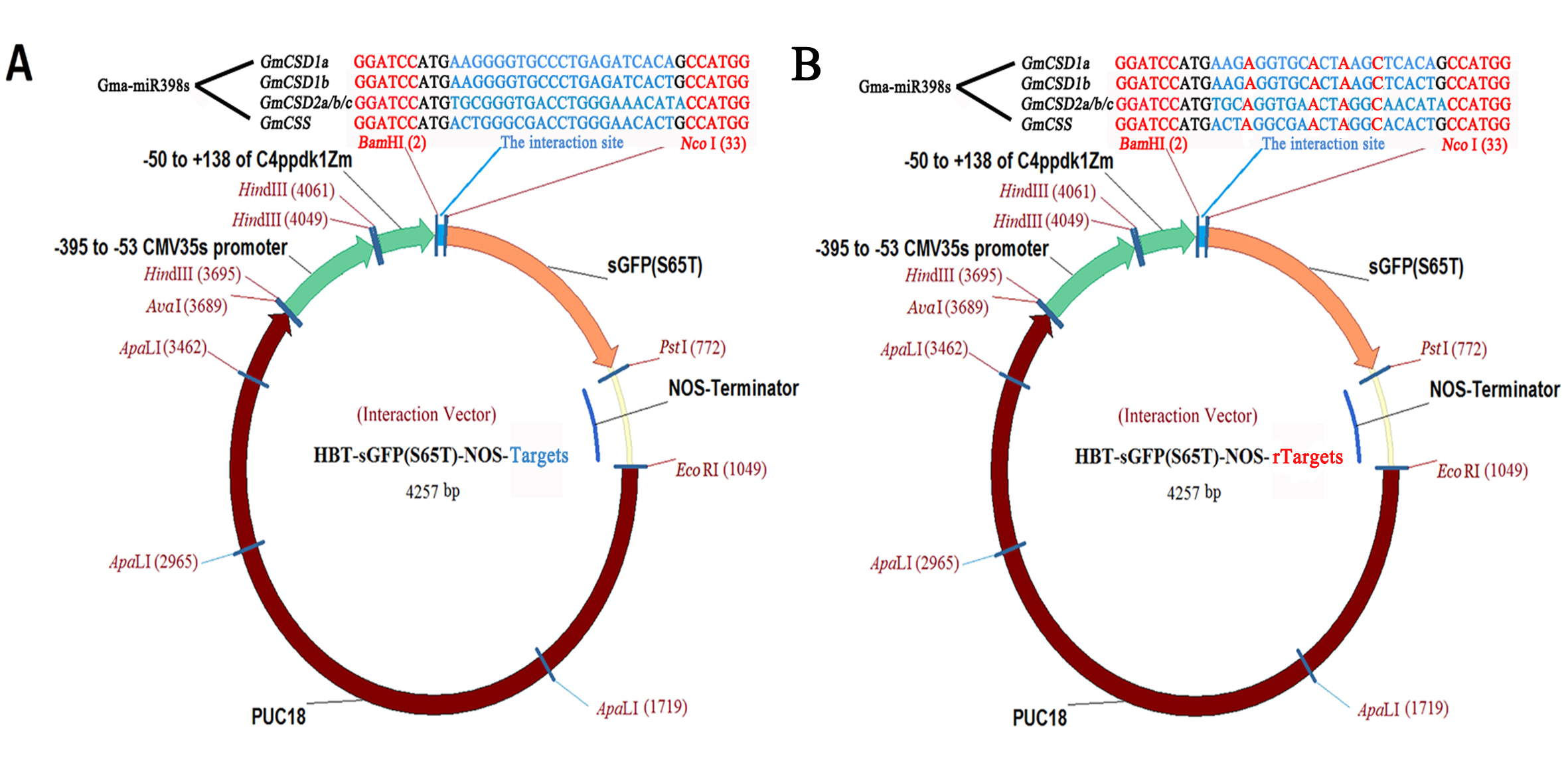

Supplement: Supplementary file 7 — Additional file 7: Figure S7. Map of the vector constructed to validate the interaction between gma-miR398c and its target genes in transiently transformed Arabidopsis mesophyll cells. (A) The HBT-sGFP(S65T)-NOS vector was used for vector construction. (B) The 21 bp conserved sequence of cleavage site was inserted into HBT-sGFP(S65T)-NOS vector to get the interaction vectors which called as HBT-sGFP(S65T)-NOS-Target. [file 12870_2020_2370_MOESM7_ESM.jpg]

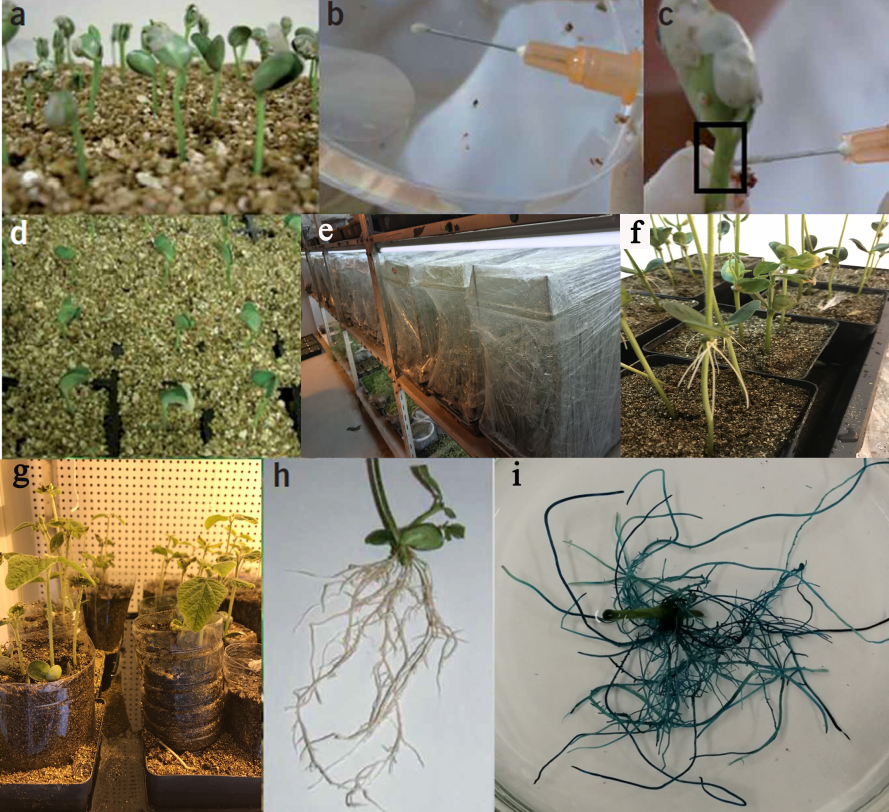

Supplement: Supplementary file 8 — Additional file 8: Figure S8. Different stages of the soybean hairy root transformation. (a) The 6-day-old seedlings of soybean. (b) Inoculation with bacterial paste. (c) Stabbing of the hypocotyl close to the cotyledonary node. (d) Growth of soybeans in pots. (e) Place a small bowl of water for 5 to 6 days to keep water. (f) Hairy roots after (e) step. (g) The wounding sites of soybean are cultivated by wet vermiculite. Soybean cultivated for about 20 days after inoculation. (h) The growth of hairy roots after the removal of the primary root. (i) Soybean hairy root GUS staining. [file 12870_2020_2370_MOESM8_ESM.jpg]

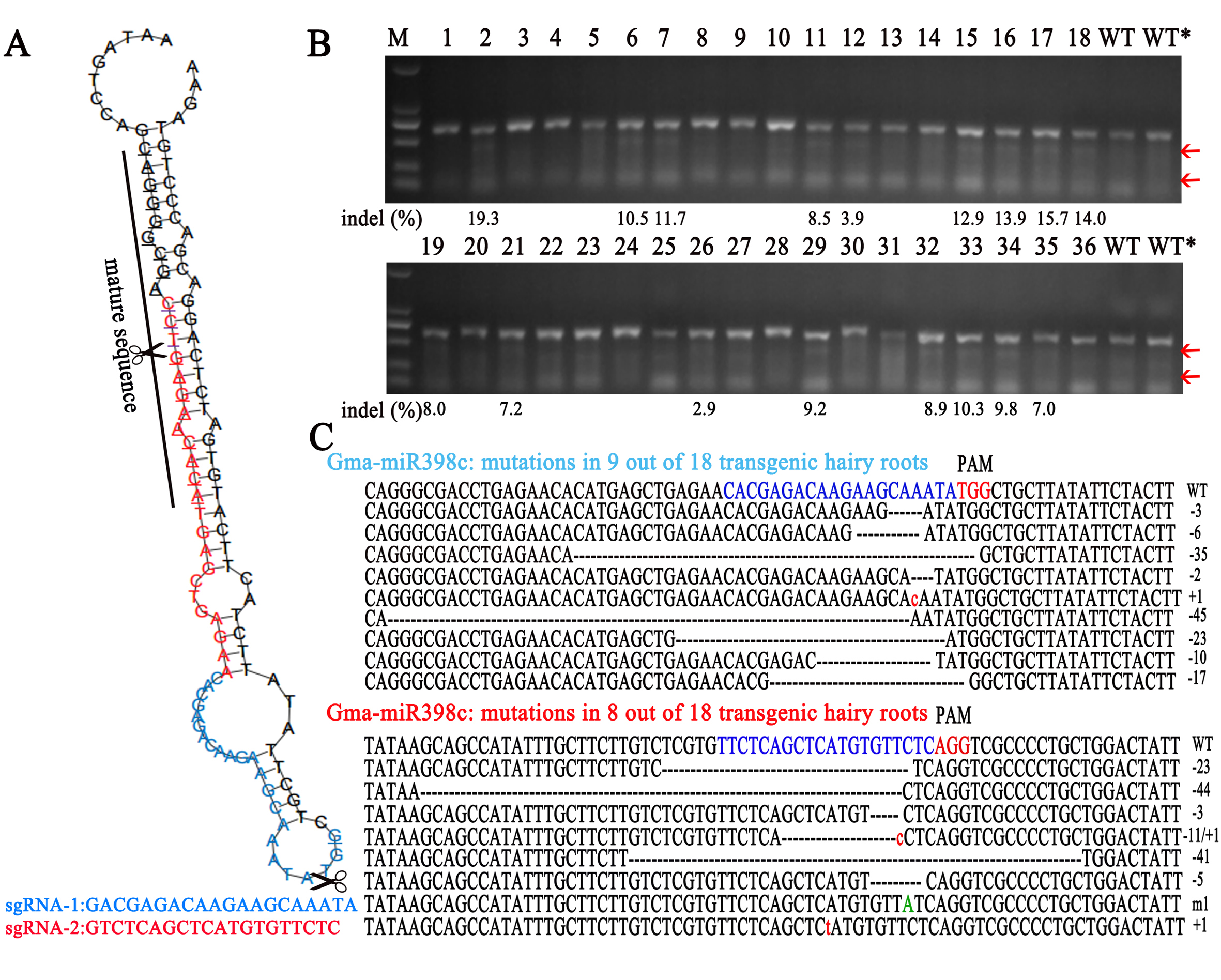

Supplement: Supplementary file 9 — Additional file 9: Figure S9. The editing system of gma-miR398c gene in soybean hairy root by CRISPR/Cas9. (A) Soybean precursor miR398c stem-loop structure and sgRNA information. (B) T7E1 enzyme digestion sgRNA-1 and sgRNA-2 editing efficiency. Lanes WT and WT*, undigested and digested wild-type controls, respectively. The red arrowhead indicates the digested bands. The numbers at the bottom of the gels indicate mutation frequencies measured according to band intensities. M, DL2000 ladder DNA marker. (C) Cloning and sequencing of the digested bands. sgRNA-1 and sgRNA-2 target site editing information. [file 12870_2020_2370_MOESM9_ESM.jpg]
