# Supplementary material for: Integration of sRNA, degradome, transcriptome analysis and functional investigation reveals gma-miR398c negatively regulates drought tolerance via GmCSDs and GmCCS in transgenic Arabidopsis and soybean
Source: BMC Plant Biol. 2020 May 5;20:190. doi: 10.1186/s12870-020-02370-y (PMC7201782; doi:10.1186/s12870-020-02370-y)
Supplement: Supplementary file 16 — Additional file 16: Table S7. Details regarding the primers used in this study. [file 12870_2020_2370_MOESM16_ESM.docx]

**Table S7. Details regarding the primers used in this study.**

| **Primers of reference genes for RT-qPCR normalization** | | | | | | | | | | | | | | | | | | | | | | |  |  |  |  |  |
| --- | --- | --- | --- | --- | --- | --- | --- | --- | --- | --- | --- | --- | --- | --- | --- | --- | --- | --- | --- | --- | --- | --- | --- | --- | --- | --- | --- |
| **Symbol** | **Accession No. (Phytozome)** | | | | | | **Primers** | | | | | | | | | | | | | | **Product**  **(bp)** | |  |  |  |  |  |
| *Actin* | Glyma.02G091900 | | | | | | F：GACCTTCAACACCCCTGCT | | | | | | | R：GTGGGAGTGCATAACCCTC | | | | | | | 143 | |  |  |  |  |  |
| *EF1b* | Glyma.14G039100 | | | | | | F：TGGTGATGAGACAGAGGAGGA | | | | | | | R：AACATCGAGAAGGACAGAAGA | | | | | | | 106 | |  |  |  |  |  |
| *Actin* | AT3G46520 | | | | | | F：GCTAACCGTGAGAAGATGAC | | | | | | | R：CTAGCATAAAGCGACAGGAC | | | | | | | 123 | |  |  |  |  |  |
| **Primers of *CSDs, CCS and other genes* for RT-qPCR analysis** | | | | | | | | | | | | | | | | | | | | | | |  |  |  |  |  |
| **Symbol** | **Accession No.**  **( Phytozome)** | | | | | | **Primers** | | | | | | | | | | | | | | **Product**  **(bp)** | |  |  |  |  |  |
| *GmCSD1a* | Glyma.03G242900 | | | | | | F：CTTCCATCCATCTTTTTCTTCTTAC | | | | | | | R：TCCCTCCTGAACGAAGTGAATA | | | | | | | 155 | |  |  |  |  |  |
| *GmCSD1b* | Glyma.19G240400 | | | | | | F：ACTCTGCTTCCCATCATCCGTC | | | | | | | R：GAAATGCGCTCCAGTTGACAGG | | | | | | | 267 | |  |  |  |  |  |
| *GmCCS* | Glyma.05G055000 | | | | | | F：CGAAAAGGGTGAGGCCTTCT | | | | | | | R：GGTGCCATCACACGTACAGA | | | | | | | 184 | |  |  |  |  |  |
| *GmCSD2a* | Glyma.11G192700 | | | | | | F：AATGGGTGTATTTCTACGGGAG | | | | | | | R：TTACCACTGCACCAGGGCTC | | | | | | | 302 | |  |  |  |  |  |
| *GmCSD2b* | Glyma.12G081300 | | | | | | F：CACAAATGGGTGTATATCAACA | | | | | | | R：ATGCTGGAGTCAAACCAACCAC | | | | | | | 305 | |  |  |  |  |  |
| *GmCSD2c* | Glyma.12G178800 | | | | | | F：CCGCCCACACTATTCTCTTT | | | | | | | R：ACCAGAAACACGAACAGTAACA | | | | | | | 173 | |  |  |  |  |  |
| *GmNod19* | Glyma.15G131300 | | | | | | F：TACCACGTGCAGGCTAAACA | | | | | | | R：GCCAATTGCGATCGTCTTGAA | | | | | | | 208 | |  |  |  |  |  |
| *GmG3Pp1* | Glyma.12G013200 | | | | | | F：GAGATGGTTGGGCACCCTTTA | | | | | | | R：CAAACAGTGAAGTAAACAAGCCAG | | | | | | | 183 | |  |  |  |  |  |
|  | Glyma.16G057300 | | | | | | F：CATTACCTGTTCCATCCTTTGTTAG | | | | | | | R：CTATAATCTGCATCCTGAGGTAAAG | | | | | | | 148 | |  |  |  |  |  |
|  | Glyma.01G136300 | | | | | | F：GGAGGAACCTCTTCATACAATCTGG | | | | | | | R：AGCATAGAACAAGTGATGAAGGC | | | | | | | 141 | |  |  |  |  |  |
|  | Glyma.03G031800 | | | | | | F：GTGAAGCAACTCAGGCCAAA | | | | | | | R：GGCCGAATAACATTGGAGAA | | | | | | | 108 | |  |  |  |  |  |
|  | Glyma.U013800 | | | | | | F：GGATTGGGAGAGTGTGTTGTCTG | | | | | | | R：CCTCAATGTCCCCCATAATCA | | | | | | | 77 | |  |  |  |  |  |
| **Primers of gma-miR398 precursors and targets for RT-qPCR analysis in Arabidopsis** | | | | | | | | | | | | | | | | | | | | | | |  |  |  |  |  |
| **Symbol** | **Accession No.**  **(miRBase)** | | | | | | **Primers** | | | | | | | | | | | | | | **Product**  **(bp)** | |  |  |  |  |  |
| Pre-miR398c | MI0017847 | | | | | | F：GGATCCTTCTACAGGGTCGTC | | | | | | | R：TGAGCTGAGAACACGAGACAAG | | | | | | | 85 | |  |  |  |  |  |
| *AtCSD1* | AT1G08830 | | | | | | F：AACCAAAGAGAGACGAAGCAAA | | | | | | | R：GCAAAGAATCAGAAGTGACAAGC | | | | | | | 166 | |  |  |  |  |  |
| *AtCSD2* | AT2G28190 | | | | | | F：GTTGTTGGAAGAGCCTTTGTGG | | | | | | | R：CGTCAAGCCAATCACACCA | | | | | | | 123 | |  |  |  |  |  |
| *AtCCS* | AT1G12520 | | | | | | F：TGGTGTATCAACGAGTATGGAGA | | | | | | | R：CCTTGAGTTTCTCTTTCTTTCCC | | | | | | | 169 | |  |  |  |  |  |
| **Primers of *GmCSDs* and *GmCCS* analysis for 5′RACE** | | | | | | | | | | | | | | | | | | | | | | |  |  |  |  |  |
| **Symbol** | **Accession No.** **(Phytozome)** | | | | | | **Primers** | | | | | | | | | | | **First round**  **PCR product**  **(bp)** | | **Second round PCR product**  **(bp)** | | |  |  |  |  |  |
| *GmCSD1a* | Glyma.03G242900 | | | | | | 3' Primer： TCCTTGCAGACCAATGATACCACAA | | | | | 3' Nested Primer：  CTCTGCCACCAGCATTTCCAGTAGTT | | | | | | 528 | | 485 | | |  |  |  |  |  |
| *GmCSD1b* | Glyma.19G240400 | | | | | |  |  |  |  |  |  |  |  |  |  |  | 520 | | 455 | | |  |  |  |  |  |
| *GmCSD2a* | Glyma.11G192700 | | | | | | 3' Primer： TCTTTAGAAGGACATTATGCTTTACAGT | | | | | 3' Nested Primer： TAATCTTCCACCAGCATTTCCAGTTG | | | | | | 373 | | 206 | | |  |  |  |  |  |
| *GmCSD2b* | Glyma.12G081300 | | | | | |  |  |  |  |  |  |  |  |  |  |  | 370 | | 199 | | |  |  |  |  |  |
| *GmCSD2c* | Glyma.12G178800 | | | | | | 3' Primer：  ACAAGCAATAGAGTGTCATTCAGTAAA | | | | | 3' Nested Primer：  AACAACATTCAACAGTAGGTTTATTCAC | | | | | | 525 | | 475 | | |  |  |  |  |  |
| *GmCCS* | Glyma.05G055000 | | | | | | 3' Primer： TATCCAAAAGTTCATAATTCTGGTGAAG | | | | | 3' Nested Primer： AATATGGTAGAGCTGATGGGCGACAA | | | | | | 445 | | 359 | | |  |  |  |  |  |
| ***Glycine max* mature miR398 gene family for stem-loop RT-qPCR** | | | | | | | | | | | | | | | | | | | | | | | |  |  |  |  |
| **Symbol** | | **Accession** | | | | **Stem-loop primer** | | | | | **Forward primer** | | | | | **Reversed primer** | | | | | | | |  |  |  |  |
| miR398a/b | | [MIMAT0001689](http://www.mirbase.org/cgi-bin/mature.pl?mature_acc=MIMAT0001689)  [MIMAT0001690](http://www.mirbase.org/cgi-bin/mature.pl?mature_acc=MIMAT0001690) | | | | GTCGTATCCAGTGCGTGTCGTGGAGTCGGCAATTGCACTGGATACGACAAGGGGT | | | | | GGGGTGTGTTCTCAGGTCA | | | | | CAGTGCGTGTCGTGGAGT | | | | | | | |  |  |  |  |
| miR398c/d | | [MIMAT0020997](http://www.mirbase.org/cgi-bin/mature.pl?mature_acc=MIMAT0020997)  [MIMAT0036343](http://www.mirbase.org/cgi-bin/mature.pl?mature_acc=MIMAT0036343) | | | | GTCGTATCCAGTGCGTGTCGTGGAGTCGGCAATTGCACTGGATACGACCAGGGGC | | | | | GGGGTGTGTTCTCAGGTCG | | | | | CAGTGCGTGTCGTGGAGT | | | | | | | |  |  |  |  |
| miR2111b/c/e/f | | MIMAT0023220  MIMAT0023221  [MIMAT0023237](http://www.mirbase.org/cgi-bin/mature.pl?mature_acc=MIMAT0023237)  MIMAT0023238 | | | | GTCGTATCCAGTGCGTGTCGTGGAGTCGGCAATTGCACTGGATACGACTAAACCT | | | | | GGGGTAATCTGCATCCTGAG | | | | | CAGTGCGTGTCGTGGAGT | | | | | | | |  |  |  |  |
| miR171n/p | | [MIMAT0023202](http://www.mirbase.org/cgi-bin/mature.pl?mature_acc=MIMAT0023202)  [MIMAT0023208](http://www.mirbase.org/cgi-bin/mature.pl?mature_acc=MIMAT0023208) | | | | GTCGTATCCAGTGCGTGTCGTGGAGTCGGCAATTGCACTGGATACGACTAAGATA | | | | | GGGGTTGAGCCGCGTCAATA | | | | | CAGTGCGTGTCGTGGAGT | | | | | | | |  |  |  |  |
| miR1520d | | [MIMAT0007379](http://www.mirbase.org/cgi-bin/mature.pl?mature_acc=MIMAT0007379) | | | | GTCGTATCCAGTGCGTGTCGTGGAGTCGGCAATTGCACTGGATACGACTTGTCAC | | | | | GGGGATCAGAACATGACACGT | | | | | CAGTGCGTGTCGTGGAGT | | | | | | | |  |  |  |  |
| **Identification of primers for transgenic Arabidopsis** | | | | | | | | | | | | | | | | | | | | | | | |  |  |  |  |
| **Symbol** | | **Forward primer** | | | | | | | **Reversed primer** | | | | | | | | **Length (bp)** | | | | | | |  |  |  |  |
| OE-vector | | CATGGTAGATCTGAGGGTAAATT | | | | | | | ACTCTGTCTGGCTTTTGGCTG | | | | | | | | 999 | | | | | | |  |  |  |  |
| OE-miR398c | | CATGAAGTAGAATATAAGCAGCCA | | | | | | | ATTGCGGGACTCTAATCATAAAAAC | | | | | | | | 433 | | | | | | |  |  |  |  |
| **The primers information of miR398 targets for subcellular localization** | | | | | | | | | | | | | | | | | | | | | | | | | | |  |
| **Symbol** | | | | | | **ID** | | | **Primer** | | | | | | | | | | | | | | **Length(bp)** | | | |  |
| pCAMBIA-1302-*GmCSD1a* | | | | | | Glyma.03G242900.2 | | | 5' Primer：GAAGATCTG ATGGTGAAGGCTGTGGCCGT | | | | | | | 3' Primer：GACTAGTTCCTTGCAGACCAATGATACC | | | | | | | 456 | | | |  |
| pCAMBIA-1302-*GmCSD1b* | | | | | | Glyma.19G240400.4 | | | 5' Primer：GAAGATCTG ATGGTGAAGGCTGTGGCAGT | | | | | | | 3' Primer：GACTAGTTCCTTGCAGACCAATGATACC | | | | | | | 348 | | | |  |
| pCAMBIA-1302-*GmCSD2a* | | | | | | Glyma.11G192700.4 | | | 5' Primer：GAAGATCTG ATGCAGCTAGCAATGGCGG | | | | | | | 3' Primer：GACTAGT TGCTGGAGTCAAACCAACCA | | | | | | | 612 | | | |  |
| pCAMBIA-1302-*GmCSD2b* | | | | | | Glyma.12G081300 | | | 5' Primer：GAAGATCTG ATGCAGCTAGCTGTGGCGG | | | | | | | 3' Primer：GACTAGTTGCTGGAGTCAAACCAACCAC | | | | | | | 612 | | | |  |
| pCAMBIA-1302-*GmCSD2c* | | | | | | Glyma.12G178800 | | | 5' Primer：GAAGATCTG ATGCAAGCAGCAATTGCAGC | | | | | | | 3' Primer：GACTAGT CACTGGACTCAAACCAACCA | | | | | | | 549 | | | |  |
| pCAMBIA-1302-*GmCSD3* | | | | | | Glyma.16G153900 | | | 5' Primer：GAAGATCTG ATGGAAGCTGCAAAGGGAAC | | | | | | | 3' Primer：GACTAGT AACAGATGACTGGAGCCCGA | | | | | | | 501 | | | |  |
| pCAMBIA-1302-*GmCCS* | | | | | | Glyma.05G055000 | | | 5' Primer：GAAGATCTG ATGGCATTTCTGAGGTCAAT | | | | | | | 3' Primer：GACTAGT GACCTTGCTAGTAACAAAATC | | | | | | | 912 | | | |  |
| HBT-*GmCSD1a* | | | | | | Glyma.03G242900.2 | | | 5' Primer：CGGGATCC ATGGTGAAGGCTGTGGCCGT | | | | | | | 3' Primer：CGGGATCCTCCTTGCAGACCAATGATACC | | | | | | | 456 | | | |  |
| HBT-*GmCSD1b* | | | | | | Glyma.19G240400.4 | | | 5' Primer：CGGGATCC ATGGTGAAGGCTGTGGCAGT | | | | | | | 3' Primer：CGGGATCCTCCTTGCAGACCAATGATACC | | | | | | | 348 | | | |  |
| HBT-*GmCSD2a* | | | | | | Glyma.11G192700.4 | | | 5' Primer：CGGGATCC ATGCAGCTAGCAATGGCGG | | | | | | | 3' Primer：CGGGATCC TGCTGGAGTCAAACCAACCA | | | | | | | 612 | | | |  |
| HBT-*GmCSD2b* | | | | | | Glyma.12G081300 | | | 5' Primer：CGGGATCC ATGCAGCTAGCTGTGGCGG | | | | | | | 3' Primer：CGGGATCCTGCTGGAGTCAAACCAACCAC | | | | | | | 612 | | | |  |
| HBT-*GmCSD2c* | | | | | | Glyma.12G178800 | | | 5' Primer：CGGGATCC ATGCAAGCAGCAATTGCAGC | | | | | | | 3' Primer：CGGGATCC CACTGGACTCAAACCAACCA | | | | | | | 549 | | | |  |
| HBT-*GmCSD3* | | | | | | Glyma.16G153900 | | | 5' Primer：CGGGATCC ATGGAAGCTGCAAAGGGAAC | | | | | | | 3' Primer：CGGGATCC AACAGATGACTGGAGCCCGA | | | | | | | 501 | | | |  |
| HBT-*GmCCS* | | | | | | Glyma.05G055000 | | | 5' Primer：CGGGATCC ATGGCATTTCTGAGGTCAAT | | | | | | | 3' Primer：CGGGATCC GACCTTGCTAGTAACAAAATC | | | | | | | 909 | | | |  |
| **Primers for the construction of CRISPR/Cas9** | | | | | | | | | | | | | | | | | | | | | | | | | |  |  |
| **Symbol** | | | | **Forward primer** | | | | | | | | | | **Reversed primer** | | | | | | | | | | | |  |  |
| sgRNA-1 | | | | GGGTTGACGAGACAAGAAGCAAATA | | | | | | | | | | AAACTATTTGCTTCTTGTCTCGTCA | | | | | | | | | | | |  |  |
| sgRNA-2 | | | | GGGTTGTCTCAGCTCATGTGTTCTC | | | | | | | | | | AAACGAGAACACATGAGCTGAGACA | | | | | | | | | | | |  |  |
| **PCR primers of genomic DNA from transgenic roots** | | | | | | | | | | | | | | | | | | | | | | | | | | | |
| **Symbol** | | | | | **Forward primer** | | | | | | **Reversed primer** | | | | | | | | | **Length (**bp**)** | | | | | | | |
| JYZ-miR398c | | | | | GATACAGGGTGTGTTTGATAGAGTG | | | | | | TACTCTTAACGGAACAAACAGACC | | | | | | | | | 705 | | | | | | | |
